# Optoelectronics Interfaces for a VLC System for UHD Audio-Visual Content Transmission in a Passenger Van: HW Design

**DOI:** 10.3390/s24175829

**Published:** 2024-09-08

**Authors:** Carlos Iván del Valle Morales, Juan Sebastián Betancourt Perlaza, Juan Carlos Torres Zafra, Iñaki Martinez-Sarriegui, José Manuel Sánchez-Pena

**Affiliations:** 1 The Photonic Displays and Applications Group (GDAF), Electronic Technology Department, University Carlos III of Madrid, Calle Butarque 15, Leganés, 28911 Madrid, Spain; jbetanco@ing.uc3m.es (J.S.B.P.); jctzafra@ing.uc3m.es (J.C.T.Z.); jmpena@ing.uc3m.es (J.M.S.-P.); 2 TECNALIA, Basque Research and Technology Alliance (BRTA), Paseo de la Castellana 200, 28046 Madrid, Spain; inaki.martinez@tecnalia.com

**Keywords:** analog-to-digital interface, digital-to-analog interface, energy harvesting, light fidelity, optical wireless communications, self-powered system, visible light communication

## Abstract

This work aims to provide the hardware (HW) design of the optoelectronics interfaces for a visible-light communication (VLC) system that can be employed for several use cases. Potential applications include the transmission of ultra-high-definition (UHD) streaming video through existing reading lamps installed in passenger vans. In this use case, visible light is employed for the downlink, while infrared light is used for the uplink channel, acting as a remote controller. Two primary components -a Light Fidelity (LiFi) router and a USB dongle—were designed and implemented. The ‘LiFi Router’, handling the downlink channel, comprises components such as a visible Light-Emitting Diode (LED) and an infrared receiver. Operating at a supply voltage of 12 V and consuming current at 920 mA, it is compatible with standard voltage buses found in transport vehicles. The ‘USB dongle’, responsible for the uplink, incorporates an infrared LED and a receiver optimized for visible light. The USB dongle works at a supply voltage of 5 V and shows a current consumption of 1.12 A, making it well suited for direct connection to a universal serial bus (USB) port. The bandwidth achieved for the downlink is 11.66 MHz, while the uplink’s bandwidth is 12.27 MHz. A system competent at streaming UHD video with the feature of being single-input multiple-output (SIMO) was successfully implemented via the custom hardware design of the optical transceivers and optoelectronics interfaces. To ensure the system’s correct performance at a distance of 110 cm, the minimum signal-to-noise ratio (SNR_min_) for both optical links was maintained at 10.74 dB. We conducted a proof-of-concept test of the VLC system in a passenger van and verified its optimal operation, effectively illustrating its performance in a real operating environment. Exemplifying potential implementations possible with the hardware system designed in this work, a bit rate of 15.2 Mbps was reached with On–Off Keying (OOK), and 11.25 Mbps was obtained with Quadrature Phase Shift Keying (QPSK) using Orthogonal Frequency-Division Multiplexing (OFDM) obtaining a bit-error rate (BER) of 3.3259 × 10^−5^ in a passenger van at a distance of 72.5 cm between the LiFi router and the USB dongle. As a final addition, a solar panel was installed on the passenger van’s roof to power the user’s laptop and the USB dongle via a power bank battery. It took 13.4 h to charge the battery, yielding a battery life of 22.3 h. This characteristic renders the user’s side of the system entirely self-powered.

## 1. Introduction

With the continual expansion of cities, commuting distances and times have notably increased. In the majority of key metropolitan areas, residents spend over an hour each day commuting to and from work via public transportation [1,2]. Moreover, the everyday usage of transportation modes such as trains, buses, and subways is on a steady annual rise. Consequently, an increasing number of people are investing a significant part of their day in transit-time that could be alternatively used for work, reading, or indulging in multimedia content.

With the substantial surge in streaming platforms in recent years, subscribers’ consumption of multimedia content, particularly on-demand movies and TV series, has shown a marked increase. This trend signifies a clear shift in consumption patterns towards more selective and demanding behaviors [3,4]. Viewers now prefer to have control over what content they watch, the manner in which they watch it, and, most crucially, when and where they watch it. In essence, contemporary viewers seek ultra-high-definition (UHD) content that they can enjoy anywhere, anytime, and on-demand.

Currently, no services offer a fully satisfying experience in transportation due to an amalgamation of business- and technology-related hindrances. The business constraints are mainly due to restrictive data consumption models dictated by telephone and Internet service providers, which is not the focal point of this research. Technologically, the main challenges present as low signal coverage, inadequate speed, and the limited bandwidth accessible to users. These issues are amplified by an increasingly crowded radio spectrum, causing interference between radio frequency waves from assorted services like telephones and other network-connected devices through wireless fidelity (WiFi). A prospective solution to these challenges could be operating within different sections of the electromagnetic spectrum, perhaps the optical spectrum.

In optical communications, a particular scenario involves the exchange of information between transmitter and receiver devices, harnessing visible and infrared (IR) light sources. This type of communication is recognized as optical wireless communication (OWC). Traditionally, due to the low cost of optoelectronic and electro-optical components, IR technology has been extensively employed within OWC. However, its usage is confined by the need to sustain low transmission powers and speeds to evade potential health hazards [5,6]. 

In recent years, visible light communication (VLC) has made considerable strides, attaining data rates of up to several Gbps. However, video transmission systems documented in the existing literature significantly trail behind these upper limits. For instance, a study proposed in [7] illustrates the concurrent transmission of audio and video signals using white and red Light-Emitting Diodes (LEDs), achieving data rates of up to 2 Mbps for video and 15 Mbps for audio over a 50 cm link, without the requirement of lenses or other optical elements to extend the distance. Similarly, ref. [8] reported the implementation of a Field-Programmable Gate Array (FPGA)-based VLC system for real-time video transmission, achieving a communication distance of up to 5 m and employing a pulse position decision algorithm to enhance transmission reliability.

Other noteworthy studies include [9], reporting a system reaching a maximum bit rate of 0.986 Mbps over an optical distance of 6 m, and [10], achieving a bandwidth of 8 MHz and an optical distance of 8 m using an avalanche photodiode (APD) as a photodetector. Furthermore, ref. [11] exhibited a maximum rate of 0.415 Mbps with video qualities ranging from 480p to 720p. A system achieving a bit rate of 7.14 Mbps over an optical link of 6.7 cm was developed in [12], whereas the system proposed in [13] managed video transmission at a rate of 0.115 Mbps over an optical link of 1 m. The research shared in [14] demonstrated a system transmitting video at a rate of up to 0.115 Mbps over a 5 m distance between the transmitter and receiver. Lastly, the proposal in [15] was based on high-powered lasers, which are infeasible for applications involving people due to potential damage to the cornea of the eyes [5] and skin [6].

Conversely, the study conducted in [16] illustrates how Orthogonal Frequency-Division Multiplexing (OFDM) modulation can be employed for boosting spectral efficiency in VLC systems, thereby achieving data rates of up to several Gbps.

A development closely related to VLC is Light Fidelity (LiFi) technology, which integrates bidirectional and multi-user communication. This technology forms wireless optical networks with seamless handover capabilities supporting user mobility. LiFi offers higher bandwidth and transmission rates compared to radio frequency (RF) technologies such as WiFi. Although LiFi cannot penetrate opaque objects, this limitation can be advantageous for applications requiring high security levels.

Our proposed optoelectronics interfaces can be utilized to provide an innovative solution for transmitting UHD multimedia content in public transportation settings. By utilizing existing reading lights in public transport vehicles, our system sets itself apart from previous studies. Moreover, to our knowledge, no work has reported a VLC system capable of transmitting UHD video using an LED as a transmitter. Additionally, our system offers the flexibility of selecting the modulation scheme—be it On–Off Keying (OOK), OFDM, or any other scheme implemented in the FPGA—by simply toggling a switch.

By employing OFDM modulation, our system can achieve data rates comparable to those reported in prior studies. Further, by leveraging high-efficiency LEDs and advanced signal processing techniques, our scheme enhances the transmission rate and improves energy efficiency and connection stability.

In this study, we conducted experimental tests in a passenger van, a specific environment that aptly represents the broader public transport context. Passenger vans, commonly used for passenger transport in many metropolitan areas, share key operational characteristics with other public transportation modes such as buses and trains. These features include constant movement, the presence of multiple users, and the need for efficient and secure communication systems.

Testing in a passenger van allowed us to evaluate the VLC technology under controlled yet realistic conditions, facilitating an accurate observation and measurement of the system’s performance. Since there is a similarity in lighting infrastructure and usage conditions, the results obtained can be extrapolated to other public transportation vehicles. Therefore, this experimental approach provides valuable and applicable information for implementing VLC systems in public transportation.

The integration of VLC in public transport offers several advantages over traditional wireless technologies. Unlike WiFi and RF, VLC is immune to electromagnetic interference, a crucial element in densely populated environments like trains and buses. Recent studies suggest that VLC can achieve higher spectral efficiency and transmission rates, reaching up to several Gbps in controlled scenarios [16]. Additionally, the ability to utilize existing lighting infrastructure significantly reduces deployment and maintenance costs. These advantages make VLC an ideal solution for enhancing user experiences in public transportation by providing a secure, fast, and reliable connection for streaming UHD multimedia content. The studies conducted in [7,8,12] also attest to the viability of VLC for real-time applications, a critical aspect of its implementation in transportation systems.

Hence, the driving force behind this study is to explore the practicality of the hardware implementation of optical transceivers and optoelectronic interfaces. The goal is to develop a VLC system that can be utilized for various use cases, including the transmission of UHD video in a passenger van.

With this in mind, a conceivable use case of this VLC system intends to leverage the reading lights pre-installed on the interior roofs of vehicles. This aims to equip public transportation with the ability to provide passengers access to UHD audio-visual content on handheld devices such as laptops, tablets, and smartphones. In order to play these audio-visual contents, users are required to connect a purpose-built transceiver or dongle to their devices’ USB port. The audio-visual content must be stored on a server that is accessible within the vehicle.

**Figure 1:**
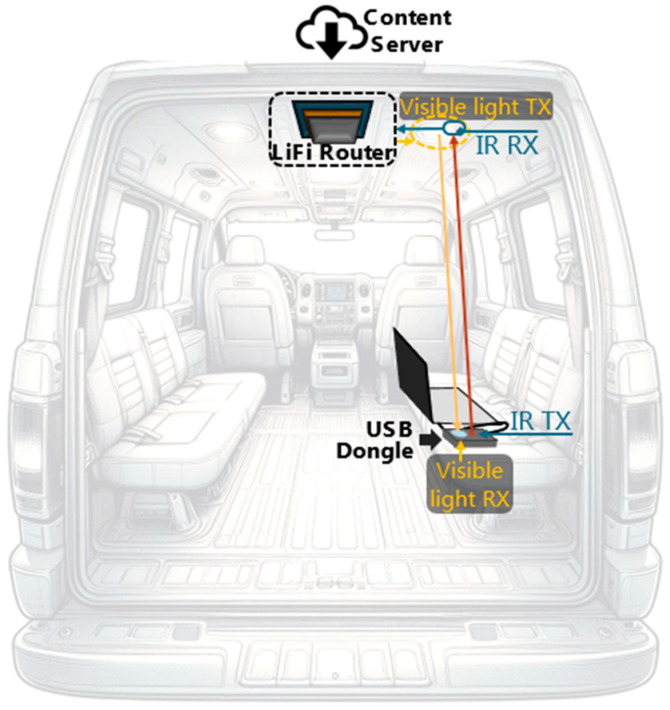
The LiFi router, located on the vehicle’s interior roof, connects to the content server via a wireless Internet connection. The USB dongle, connected to the user’s portable device, receives and plays the audio-visual content as described.

The proposed system encompasses two principal components: (i) a LiFi router and (ii) a USB dongle, as graphically represented in Figure 1.

In the ensuing sections, the components and methods utilized in the development of the optoelectronic interfaces will be outlined. Initially, the proposed optoelectronic interface system will be detailed, covering its application in both the downlink and uplink. This will be followed by a more thorough elaboration on the design and implementation of the system components, including the LED and photodiode (PD) driver. Subsequently, we will describe the experimental set-up, testing procedure, and the system’s deployment in a passenger van. Finally, the most representative measurements characterizing the system will be presented, leading to the final conclusions.

## 2. Materials and Methods

### 2.1. Description of the Proposed System

The communication system is bidirectional, featuring both a downlink and an uplink. The downlink is based on VLC technology, meaning it employs white light to transmit audio-visual content from the LiFi router to the universal serial bus (USB) dongle. Conversely, the uplink uses infrared light to send control commands, similar to those found in TV remote controls, from the end user’s device to the LiFi router. The infrared light does not interfere with the white light used in the downlink, as they operate at different wavelengths [17,18,19]. Both the LiFi router and the USB dongle utilize this bidirectional communication, making use of both the downlink and uplink for efficient data transmission in both directions.

The van wirelessly receives data streaming through packets from the content server via a mobile network or WiFi. The LiFi router then takes these packets, which are digital signals, and converts them into analog signals. The analog signals modulate the white-light LED, which emits optical signals [20]. Thus, the visible-light emitter serves a dual purpose: (i) providing illumination to the indoor vehicle and (ii) transmitting modulated information to the visible-light receiver located at the USB dongle.

The USB dongle receiver detects the visible optical signals, which performs the reverse function of the LiFi router by transforming the visible optical signals back into analog signals, and then the analog signals are converted into digital signals and these digital signals are joint to form the packets which are transmitted through the USB port to the end user device, such as a laptop, tablet, or smartphone. In the reverse direction, the user can send commands such as rewind, forward, play, pause, or stop to the LiFi router through the infrared uplink. This separation of the downlink and uplink into different wavelengths prevents interference [17,21], establishing a bidirectional communication system.

**Figure 2:**
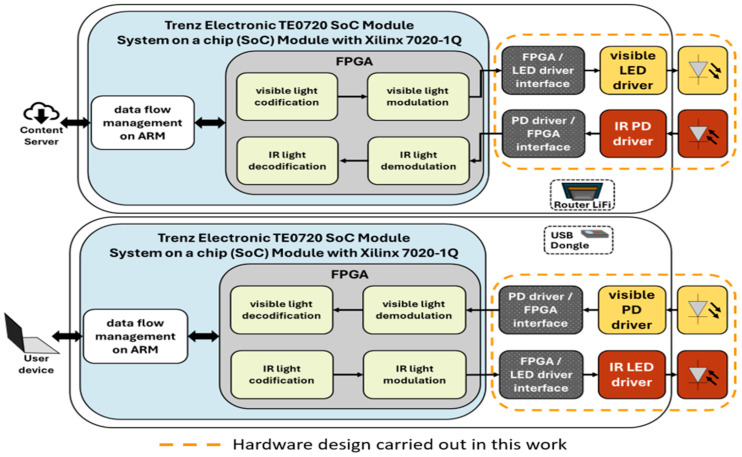
Block diagram of the VLC proposed system.

The block diagram of the system is shown in Figure 2. The diagram includes the LiFi router and the USB dongle, which both consist of optical components such as an LED, PD, electronic drivers, and interfaces for the uplink and downlink, all depicted in blue, yellow and orange colors, respectively. Additionally, the advanced RISC machine (ARM) and FPGA blocks are integrated into a Trenz Electronic TE0720 system on a chip (SoC) module [22]. The ARM block handles firmware development, while the FPGA block encompasses virtual hardware. 

The ARM block is responsible for memory management processes and implementing physical (PHY) and medium access control (MAC) layers to efficiently manage data flow and its corresponding frames. The FPGA block manages the encoding and decoding of visible and infrared light signals, as well as their modulation and demodulation. Although the ARM and FPGA blocks contribute to the overall application, they are not part of the hardware development of this work. 

On the router LiFi side, the FPGA connects to the visible LED driver through the FPGA/LED driver interface, and the IR PD driver connects to the FPGA through the PD driver/FPGA interface. The router LiFi connects to the content server via a mobile network or WiFi. The server provides the UHD audio-visual content to the system.

On the USB dongle side, the visible PD driver connects to the FPGA through the PD driver/FPGA interface, and the FPGA connects to the IR LED driver through the FPGA/LED driver interface. The USB dongle connects to the user’s device via the USB port of its mobile device.

The visible/IR LED drivers establish the bias point of the LED and stimulate it with modulated signals for emission. For the visible LED, the LED driver sets the illumination level, while the visible/IR PDs respond to variations in visible/IR light radiation, converting them into electrical signals. The PD drivers amplify the signal received by the PDs to the desired level.

To drive both the lighting LED and the IR LED, an alternating input signal is required. When implementing OFDM modulation, the generated signal has two components: (i) quadrature and (ii) phase. This requires a stage in the digital-to-analog conversion process to combine these two complex signals into a single real signal. We implemented Hermitian symmetry in the digital stage of the inverse fast Fourier transform (IFFT) to minimize the number of analog matching stages. This approach greatly simplifies the necessary hardware, although it comes at the cost of reduced system bandwidth. After a comprehensive cost–benefit analysis, this solution was chosen. It can provide the required system performance while minimizing the hardware components needed, thereby significantly reducing the overall system cost.

### 2.2. Definition and Design of the System’s Components

#### 2.2.1. LED Driver

The LUW-CN7N-KYLX-EMKM OSRAM LED [23] was selected for the visible-light downlink. This LED was bought on https://eu.mouser.com/ (accesed on 5 September 2024). This high-power LED boosts the detection of the transmitted signal, offering improved optical link reach distance compared to other LEDs, like the LUXEON 3020 LED [24]. 

The bias point of the LUW-CN7N-KYLX-EMKM LUW OSRAM LED was established at a forward voltage of 3.27 V and a forward current of 120 mA, setting an illumination level of around 75,000 lx. The LED’s cut-off frequency at −3 dB measured a value of f_−3dB_ = 1.8 MHz. 

Given the application requires around a 10 MHz of bandwidth for the visible downlink, it is critical to obtain the LED’s equivalent electric circuit to try to enhance its bandwidth.

The procedure detailed in [25] was conducted to characterize the ultra-white LUW-CN7N-KYLX-EMKM OSRAM LED in alternating current (AC) [23]. This was carried out to determine the parameters of the simplified equivalent electric circuit of the LED, yielding the following values: R_d_ = 81 mΩ, R_s_ = 26.88 Ω, and C_j_ ≃ 1.09 μF.

**Figure 3:**
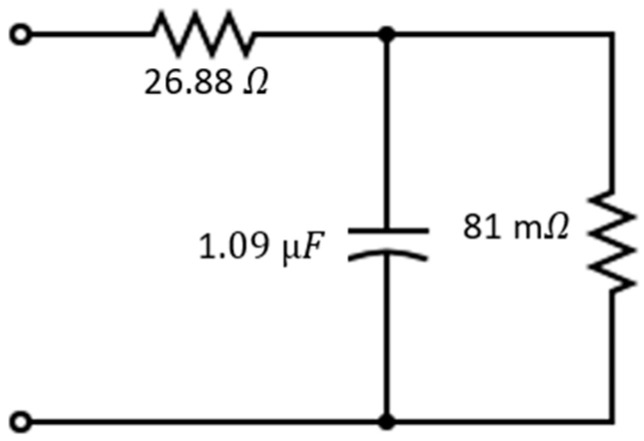
Equivalent simplified circuit of the LUW-CN7N-KYLX-EMKM OSRAM LED for AC.

The simplified equivalent electric circuit of the LUW-CN7N-KYLX-EMKM OSRAM LED, displayed in Figure 3, was simulated using the LTSpice XVII simulator software for the analog electronic circuit simulator. The simulation results showed that the cut-off frequency at −3 dB was f_−3dB_ = 1.78 MHz, which closely aligns with the measured bandwidth of f_−3dB_ = 1.8 MHz, thereby validating the proposed simplified equivalent circuit shown in Figure 3.

**Figure 4:**
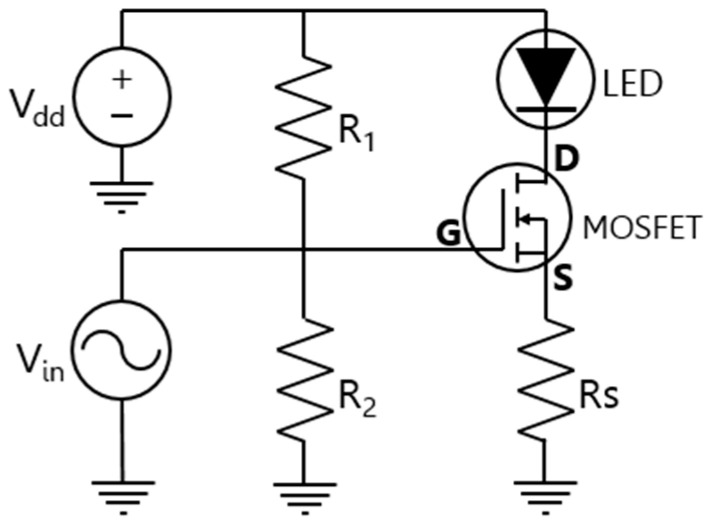
LED driver based on a division voltage for n-type MOSFET.

Before the design of the LED stage to increase its bandwidth, the LED driver was implemented based on a voltage division bias circuit developed through an n-type MOSFET transistor, as illustrated in Figure 4. The alternating current controls the transistor gate, thereby switching the LED.

The n-type MOSFET TN0110N3-G [26] was chosen to control the current flow through the LED using the resistor R_S_. This transistor was manufactured by Microchip Technology and bought on https://eu.mouser.com/ (accessed on 5 September 2024). To achieve this, the MOSFET must be in the active region. This can be ensured by confirming that the voltages V_GS_ and V_DS_ exceed certain threshold values defined in the MOSFET’s datasheet, specifically, V_GS_ > 2 V and V_DS_ > 2 V. It is worth noting that the LED driver can be tuned to adapt if the LED is replaced with another model. 

To set the bias point of the circuit, a DC source defined as V_dd_ is required to supply voltage to the LED and maintain the MOSFET in the active region. V_in_ is linked with the AC generator signal, which is short-circuited for the DC analysis performed for the bias point study.

After analyzing the voltage division bias circuit for the MOSFET transistor TN0110N3-G with a DC voltage supply of V_dd_ = 12 V, the LED’s bias point was determined as I_LED_ = 120 mA and V_LED_ = 3.27 V. The values of the passive elements in the circuit were eventually calculated as R_1_ = 10 kΩ, R_2_ = 5 kΩ, and R_S_ = 5.5 Ω.

Equalization aims to balance the signal energy in specific frequency bands to stabilize the transfer function and enhance bandwidth. By adding an equalizer block, the objective is to accomplish a response as uniform as possible within the band of interest.

**Figure 5:**
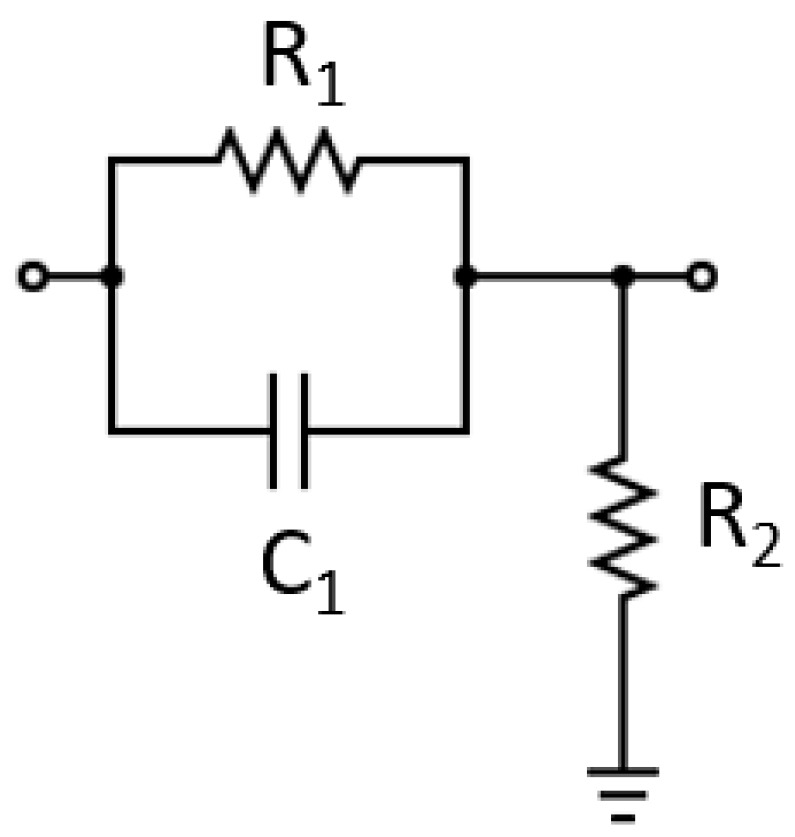
Phase-advance equalizer.

A review of the relevant literature revealed a straightforward passive equalizer circuit that introduces a pole and a zero to the system, achieving pole–zero compensation. This type of circuit, known as a phase-advance equalizer, is depicted in Figure 5.

The phase-advance equalizer can be fine-tuned to introduce a zero at the point where the LED pole is located and a pole at a further point, subsequently defining the new bandwidth. Such tuning can be achieved by adjusting the values of passive elements. It is understood that the LED transfer function corresponds to a low-pass signal, where the pole frequency has been simplified to ${LED}_{pole}$ in Equation (1). The parameters *R_d_* = 81 mΩ and *C_j_* = 1.09 μF belong to the simplified equivalent circuit of the LED and were previously calculated and are shown in Figure 3.

(1) $$H_{LED}\left(s\right)=\left(\frac{1}{R_{d}\cdot C_{j}}\right).\left(\frac{1}{s+s_{LED\_pole}}\right)$$

The transfer function of the phase-advance equalizer is represented by Equation (2), where ${equ}_{zero}\;$ denotes the zero frequency and ${equ}_{pole}$ indicates the pole frequency.

(2) $$H_{equ}\left(s\right)=\left(\frac{R_{2}}{R_{1}+R_{2}}\right).\left(\frac{s+s_{equ\_zero}}{s+s_{equ\_pole}}\right)$$

By integrating the equalizer module into the LED driver, the resultant transfer function is outlined in Equation (3).

(3) $$H_{LED\_driver}\left(s\right)=\left(\frac{1}{R_{d}\cdot C_{j}}\right)\cdot \left(\frac{R_{2}}{R_{1}+R_{2}}\right)\cdot \left(\frac{1}{s+s_{LED\_pole}}\right)\cdot \left(\frac{s+s_{equ\_zero}}{s+s_{equ\_pole}}\right)$$

As stated earlier, the zero frequency of the equalizer was set at the LED’s pole frequency, while the pole frequency of the equalizer was adjusted to obtain the desired −3 dB cutoff frequency in the resultant function. This adjustment led to Equation (4).

(4) $$H_{LED\_driver}\left(s\right)=\left(\frac{1}{R_{d}\cdot C_{j}}\right)\cdot \left(\frac{R_{2}}{R_{1}+R_{2}}\right)\cdot \left(\frac{1}{s+s_{equ\_pole}}\right)$$

**Figure 6:**
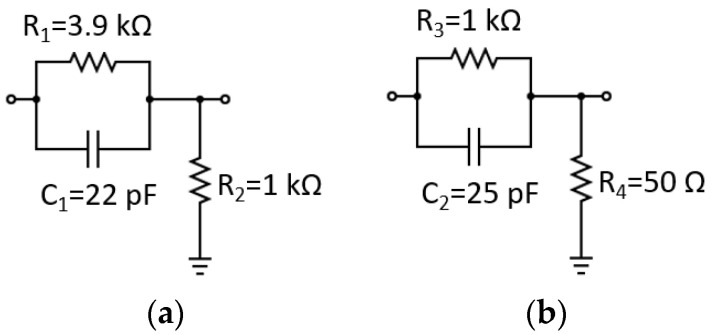
Phase-advance equalizer implemented for (**a**) the first stage for a pole located at 1.8 MHz, and (**b**) the second stage for a pole located at 9 MHz.

With ${LED}_{pole\_1}$ = 1.8 MHz, the zero frequency of the equalizer (${equ}_{zero\_1})$ and the pole frequency of the equalizer $\left({equ}_{pole_{1}}\right)$ were set to 1.8 MHz and 9 MHz, respectively. This configuration resulted in *R*_1_ = 3.9 kΩ, *C*_1_ = 22 pF, and *R*_2_ = 1 kΩ, as displayed in Figure 6a. Similarly, a second equalizer was proposed to increase the bandwidth from 9 MHz to 18 MHz. With ${LED}_{pole\_2}$ = 9 MHz, the zero frequency of the equalizer (${equ}_{zero\_2})$ and the pole frequency of the equalizer $\left({equ}_{pole_{2}}\right)$ were set to 9 MHz and 18 MHz, respectively. This set-up resulted in *R*_3_ = 1 kΩ, *C*_3_ = 25 pF, and *R*_4_ = 50 Ω, as shown in Figure 6b. 

**Figure 7:**
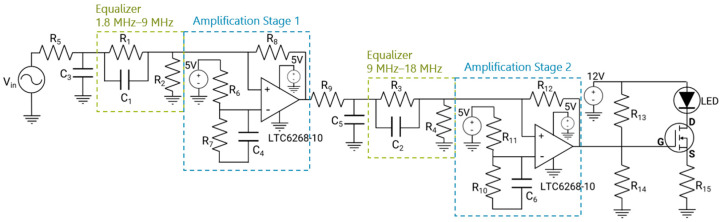
LED driver implemented with 2 equalization and 2 amplification stages.

After incorporating two equalization stages, a noticeable decrease in amplitude was observed. To rectify this effect, two amplification stages were introduced to balance the LED driver’s frequency response, as displayed in Figure 7. The components selected for the LED driver are listed in Table 1. The datasheets for the MOSFET transistor TN0110N3-G and the amplifier LTC6268-10 can be found in [26], respectively.

**Figure 8:**
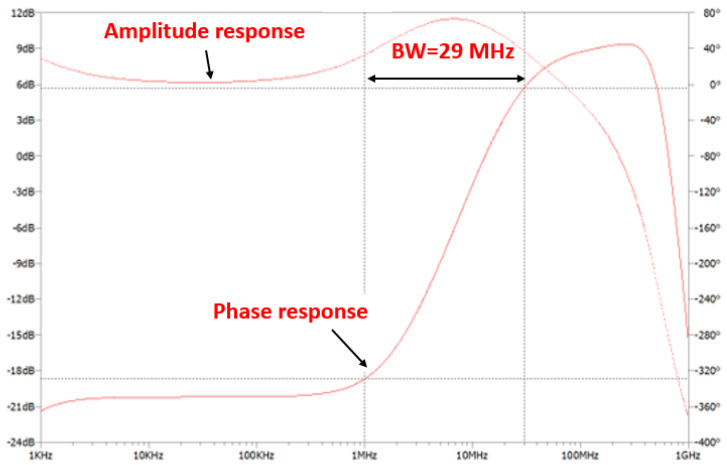
Simulated frequency response of the proposed LED driver based on two amplification and two equalization stages.

The simulation results of this circuit are demonstrated in Figure 8, which reveal the frequency response of the LED driver with two equalization and two amplification stages. The circuit attained a −3 dB cut-off frequency of roughly 29 MHz.

The frequency and phase response of the visible optical channel were measured using a Moku frequency response analyzer [27]. This device enables the measurement of both the module and phase of the optical channel’s frequency response. The analyzer operates by sweeping a sinusoidal signal across an adjustable frequency range from 10 mHz to 20 MHz, and it can apply a DC bias of up to ±5 V.

**Figure 9:**
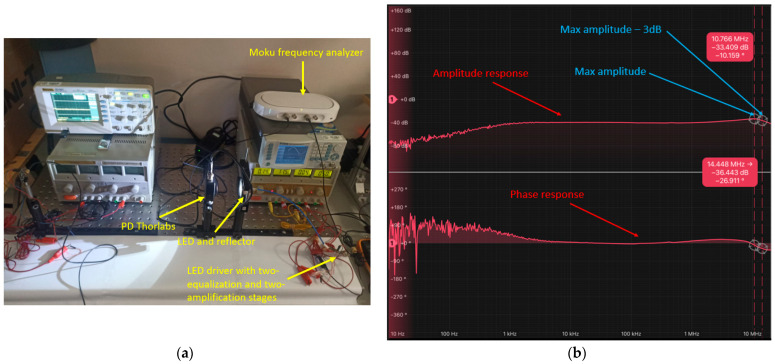
(**a**) Set-up implemented to take the frequency response measurement of the LUW-CN7N-KYLX-EMKM OSRAM LED and its LED driver based on 2 equalization and 2 amplification stages; (**b**) frequency response measurement of the LUW-CN7N-KYLX-EMKM OSRAM LED and its LED driver based on 2 equalization and 2 amplification stages.

The measurement set-up involved positioning the LUW-CN7N-KYLX-EMKM OSRAM LED and the Thorlabs PDA10A2 PD [28] at a distance of 9 cm without any lens on the Thorlabs PDA10A2 PD (as depicted in Figure 9a). The measurement was performed under an illuminance of 30,000 lx on the Thorlabs PDA10A2 PD, with the illumination level measured using a MAVOLUX 5032C USB luxmeter [29], manufactured by Gossen and bought on https://www.pce-iberica.es/medidor-detalles-tecnicos/instrumento-de-radiacion/luxometro-mavolux.htm (accessed on 5 September 2024). The measured frequency response, which includes both the magnitude and phase, is illustrated in Figure 9b.

The bandwidth achieved by the LUW-CN7N-KYLX-EMKM OSRAM LED and its LED driver based on 2 equalization and 2 amplification stages was 14.45 MHz, which complies with the 10 MHz requirement of the VLC channel.

For the IR uplink, the IR HSDL-4250 LED [30] was selected, which is a high-power Aluminum Gallium Arsenide (AlGaAs) LED. This IR LED was manufactured by Avago and bought on https://mouser.com/ (accessed on 5 September 2024). It is optimized for high-speed IR communications and efficiency at emission wavelengths of 870 nm and presents a radiant on-axis intensity of 180 $\frac{mW}{sr}$. The measured bandwidth of the IR HSDL-4250 LED reached 2 MHz.

**Figure 10:**
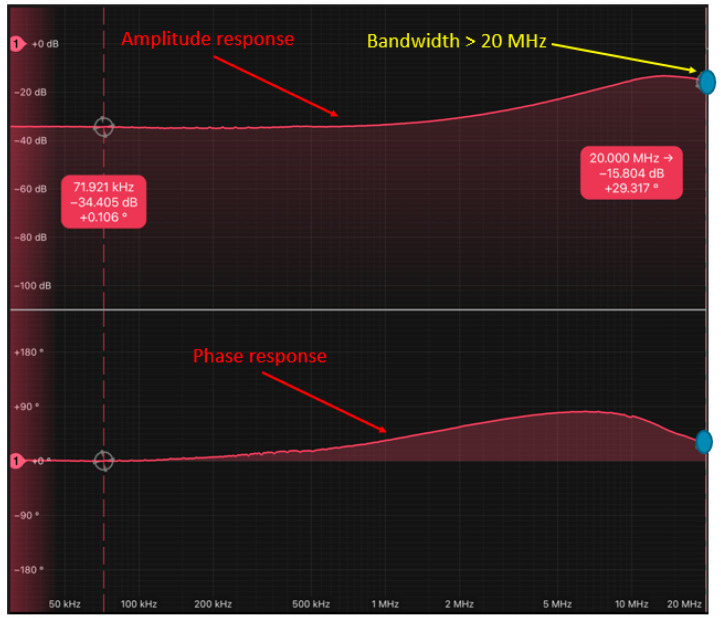
Frequency response measurement of the IR HSDL-4250 LED and its LED driver based on 2 equalization and 2 amplification stages.

Given this, the previously mentioned LED driver, encompassing 2 equalization and 2 amplification stages, was utilized for the uplink. Consequently, the bandwidth of the IR HSDL-4250 LED and its LED driver, based on 2 equalization and 2 amplification stages, exceeded 20 MHz, as demonstrated in Figure 10. This effectively complies with the 10 MHz requirement of the IR channel.

#### 2.2.2. PD Driver

In designing the PD driver, it was necessary to ensure that a bandwidth of at least 10 MHz was achieved to comply with the VLC channel requirement. We selected the OSRAM PIN BP 104 S photodiode [31] due to its optimal balance between the active area and response time. This PD was bought on https://mouser.com/ (accessed on 5 September 2024). The circuit was implemented using a trans-impedance amplifier with the LTC6268-10 amplifier [32], which was chosen for its 4 GHz gain–bandwidth (GBW) product and suitable supply voltage range. The receiver circuit was powered by a 5 V voltage supply, slightly below the maximum allowable voltage supply of the LTC6268-10 amplifier, which is 5.5 V. The PIN BP 104 S OSRAM photodiode [31] was reverse-biased at V_PD_ = V_−_ = −2.5 V to operate in the photoconductive mode. Additionally, a voltage divider was incorporated at the amplifier’s positive input to set the voltage to V_+_ = 2.5 V. As the amplifier’s input voltage range depends on the input voltages V_+_ and V_−_, the input voltage range is from −2.7 V to 2.7 V.

**Figure 11:**
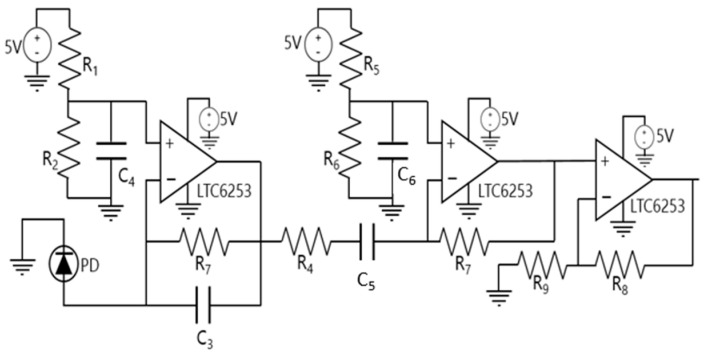
PD driver implemented with three amplifier stages.

Thus, by adding a voltage divider at the positive input of the amplifier, the input signal range permitted by the amplifier is increased. The gain of the first stage is 15 kΩ, while the gains of the second and third stages are 5 and 9, respectively. The second and third stages are based on the LCT6253 amplifier due to its GBW of 720 MHz [33]. The schematic design of the PD driver is depicted in Figure 11. The components selected for the PD driver are listed in Table 2. The datasheets of the LTC6253 amplifier can be found in [33]. 

**Figure 12:**
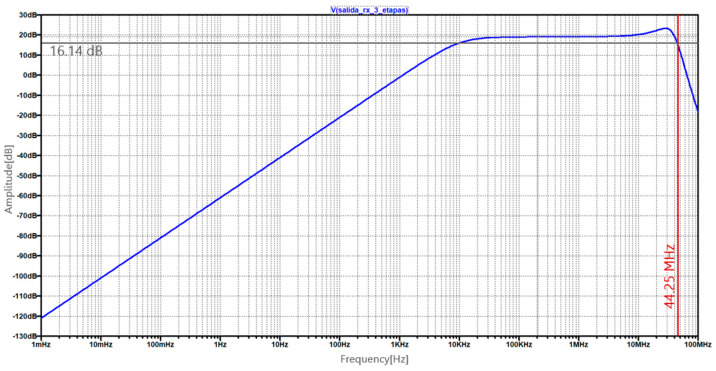
Simulated frequency response of the PD driver implemented with three amplifier stages.

Subsequently, the three-stage amplification PD driver was simulated in LTSpice, resulting in a simulated bandwidth of 44.25 MHz, as shown in Figure 12.

**Figure 13:**
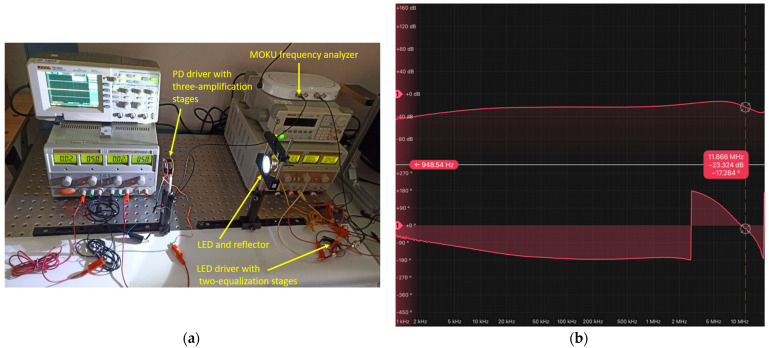
(**a**) Set-up of the frequency response of the PD driver implemented with three amplifier stages using the LUW-CN7N-KYLX-EMKM OSRAM LED; (**b**) frequency response of the PD driver implemented with three amplifier stages using the LUW-CN7N-KYLX-EMKM OSRAM LED.

The frequency and phase response of the visible optical channel were measured using the Moku frequency response analyzer. The measurement was conducted at a distance of 50 cm between the LUW-CN7N-KYLX-EMKM OSRAM LED and the three-stage PD driver, without utilizing any lens on the implemented PD driver, as depicted in Figure 13a. Additionally, the measurement was performed with an illuminance level of 500 lx on the PIN BP 104 S OSRAM PD, as recorded using the MAVOLUX 5032C USB luxmeter.

The transfer function of the three-stage PD driver is illustrated in Figure 13b, demonstrating that the measured bandwidth attained 11.66 MHz when using the LUW-CN7N-KYLX-EMKM OSRAM LED.

The measurements confirmed that the receiver circuit had an appropriate gain and aligned with the expected values. The amplitude of the received signals was 500 mV_pp_ at a distance of 50 cm between the receiver and transmitter, as evident from the 11 MHz frequency sinusoidal signal received.

**Figure 14:**
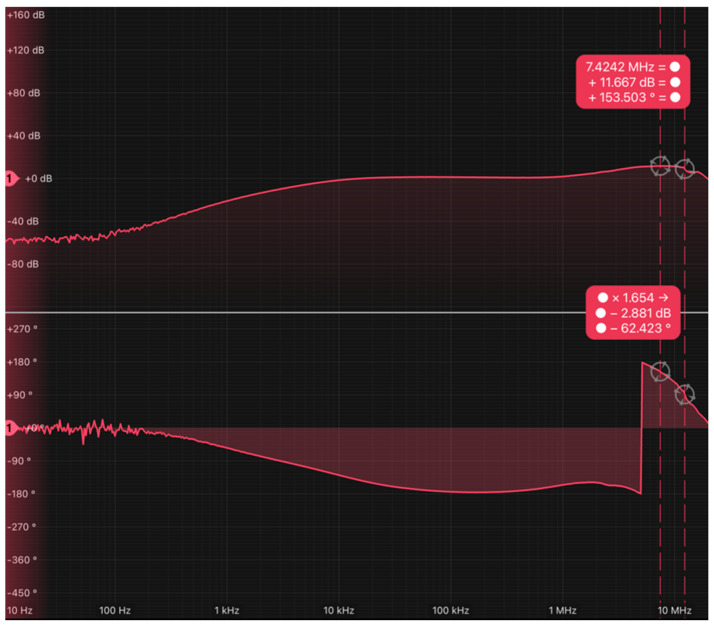
Frequency response of the PD driver implemented with three amplifier stages using the IR HSDL-4250 LED.

Given the BP 104 S OSRAM PD has a response range from 400 nm to 1100 nm, and the IR HSDL-4250 LED emits at 870 nm, it was decided to use the BP 104 S OSRAM PD and the PD driver based on three amplification stages for the IR uplink. As shown in Figure 14, the bandwidth of the BP 104 S OSRAM PD and the PD driver based on three amplification stages using the IR HSDL-4250 LED reached 12.27 MHz. 

#### 2.2.3. Internal Interfaces to Connect FPGA/LED Driver and FPGA/PD Driver

To interconnect the various subsystems, an internal interface design was implemented. This interface serves two purposes: (i) it connects the FPGA with the LED driver, and (ii) it links the PD driver with the FPGA; these connections facilitate both the downlink and uplink, respectively.

Purpose (i), the connection of the FPGA with the LED driver, requires an interface in the LiFi router to link the FPGA with the visible LED driver for the downlink. Additionally, at the USB dongle side, the interface connects the FPGA with the IR LED driver for the uplink.

**Figure 15:**
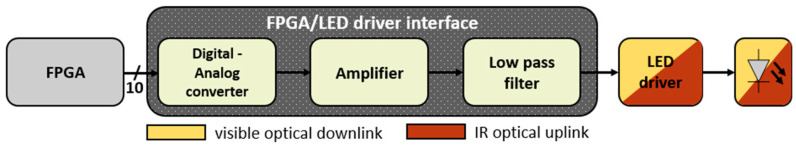
Block diagram of the FPGA/LED driver interface.

Each component that constitutes the FPGA/LED driver interface is detailed in Figure 15 and described further below.

The digital-to-analog converter (DAC) has 10 parallel data input bits connected to the output of the FPGA data bus, as illustrated in Figure 15. The DAC clock frequency is determined by the sampling frequency. We assigned a sampling frequency of 40 MHz to the DAC clock. This value was selected based on the achieved bandwidth (*BW*) of 10 MHz for each optical channel. As such, it fulfils the Nyquist criteria, which mandates the converter’s sampling frequency (*f_sampling_*) to be at least double the signal bandwidth, as outlined in Equation (5):

(5) $$f_{sampling}\;\geq \;2\cdot (BW)$$

An amplifier is employed to convert the differential output of the DAC into a single-ended output, with a gain of 2 $\frac{V}{V}$. The amplifier’s GBW product amounts to 350 MHz.

Finally, a low-pass filter is utilized to eliminate unwanted frequency components, with a cut-off frequency set at 30 MHz.

For purpose (ii), which entails connecting the PD driver to the FPGA, the interface is implemented akin to the first configuration. This interface is required in the USB dongle to interlink the PD driver and the FPGA for the downlink. Furthermore, it is necessary in the LiFi router to connect the PD driver and the FPGA for the uplink.

**Figure 16:**
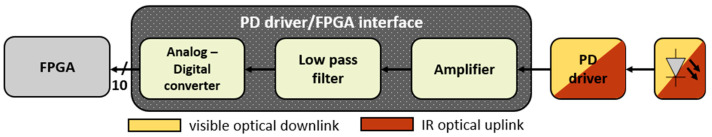
Block diagram of the PD driver/FPGA interface.

Each constituent block of the PD driver/FPGA interface is depicted in Figure 16 and described further below.

An amplifier is used to convert the single-ended signal from the PD driver into a differential signal, which is then connected to the reference inputs of the analog-to-digital converter (ADC). The amplifier has a gain of 2, and the GBW product is 350 MHz.

A low-pass filter is implemented to eliminate frequency components outside the BW of interest, with the cut-off frequency set at 30 MHz.

Considering that the system encompasses both downlink and uplink channels, the same interface design was replicated for each side of the visible and IR optical links.

##### Interface Module for FPGA/LED Driver Connection

**Figure 17:**
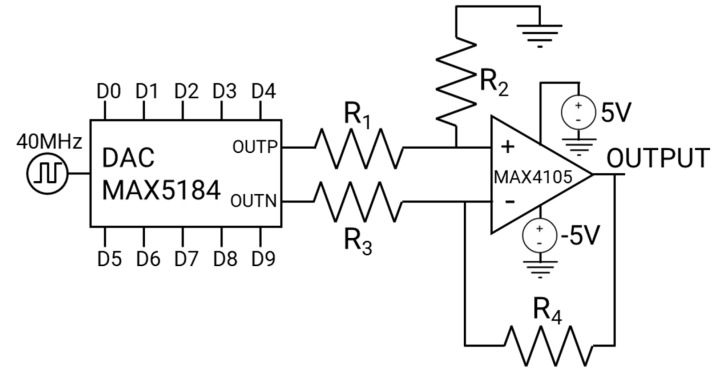
Schematic design of the FPGA/LED driver connection.

In order to connect the FPGA pinouts with the LED driver, a digital-to-analog conversion was necessary because the LED is modulated using an analog signal [20]. For this task, the MAX5184 DAC was selected due to its 10-bit resolution and ability to handle clock signals up to 40 MHz. This DAC was manufactured by Analog Devices and bought on https://mouser.com/ (accessed on 5 September 2024). This converter features two differential outputs, each with an amplitude of 400 mV_pp_ [34]. These outputs were then combined into a single-ended signal using the MAX4105 high-speed operational amplifier [35]. This amplifier was manufactured by Analog Devices and bought on https://mouser.com/ (accessed on 5 September 2024). Furthermore, the amplitude of the single-ended signal was increased to 800 mV_pp_, as illustrated in Figure 17.

We opted for the TXO crystal oscillator to generate the clock signal, which produces a stable 40 MHz square wave. This oscillator is renowned for its stability and is frequently utilized in wireless Internet of Things (IoT) applications employing the LoRa WAN protocol [36]. The crystal oscillator is integrated into the interface and can be connected to the MAX5184 DAC via a jumper. Alternatively, the clock signal can be generated from the Xilinx Zynq 7020 FPGA [37], which is integrated into the TE0720 SoC, and connected to the MAX5184 DAC [34] by adjusting the jumper’s position.

Considering that the output impedance of *OUT_P_* and *OUT_N_* is 400 Ω [34], the resistors *R*_1_ and *R*_3_ were set to 402 Ω, which is the closest standard value to 400 Ω. By analyzing the circuit loops depicted in Figure 17, we derived the following equations:

(6) $$\frac{V_{OUTP}-V_{+}}{R_{1}}=\frac{V_{+}}{R_{2}}$$

(7) $$\frac{V_{OUTN}-V_{-}}{R_{3}}=\frac{V_{-}-V_{O}}{R_{4}}$$

By defining $R_{2}=2{\cdot R}_{1}$ and $R_{4}=2\cdot R_{2}$, the output voltage assumes the following value:

(8) $$V_{O}=2\cdot (V_{OUTP}-V_{OUTN})$$

We selected *R*_2_ = 1 kΩ and *R*_4_ = 1 kΩ. The output is connected to the input of the LED driver circuit for signal modulation. The components selected for the connection between the FPGA/LED driver are listed in Table 3. The datasheets of the MAX5184 DAC, the MAX4105 amplifier, and the TXO crystal oscillator can be found in [34,35,36], respectively.

##### Interface Module for PD Driver/FPGA Connection

**Figure 18:**
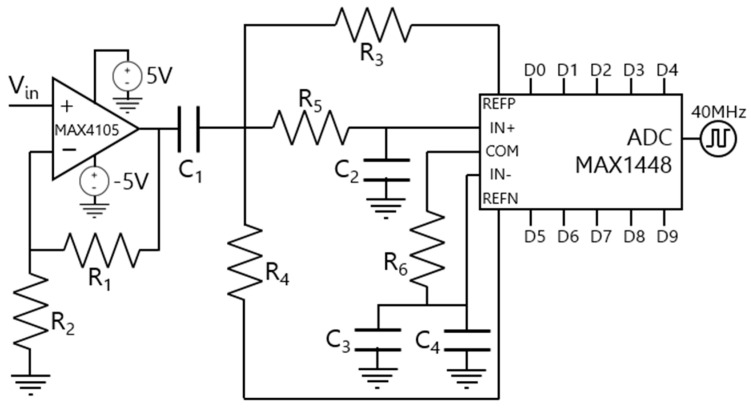
Schematic design of the PD driver/FPGA connection.

For the PD driver/FPGA interface, an analog-to-digital converter (ADC) is needed to transform the analog signal from the receiver into a 10-bit digital signal. The MAX1448 ADC [38] was chosen for its 10-bit resolution and ability to operate with clock signals of up to 80 MHz. This ADC was manufactured by Analog Devices and bought on https://mouser.com/ (accessed on 5 September 2024). Though this design employs a 40 MHz clock signal, also used by the MAX5184 DAC [34]. The implemented design, which includes the MAX4105 high-speed amplifier [35], is depicted in Figure 18.

We chose the internal reference mode [38] for the operation of the MAX1448 ADC. In this mode, the highest reference (*REF_P_*), lowest reference (*REF_N_*), and common-mode voltage range (*COM*) terminals operate as outputs, with the following values:

(9) $${REF}_{P}=\frac{V_{DD}}{2}+\frac{V_{REFIN}}{4}$$

(10) $${REF}_{N}=\frac{V_{DD}}{2}-\frac{V_{REFIN}}{4}$$

(11) $$COM=\frac{V_{DD}}{2}$$

In this context, *V_DD_* = 3 V signifies the digital supply voltage of the ADC, and *V_REFIN_* = 2 V represents the internal scale value for the chosen operating mode. As a result, the MAX1448 ADC [38] is configured to convert analog amplitude signals ranging between −1 V and 1 V.

Pertaining to the circuit implementation shown in Figure 18, the first stage entails a conditioning phase employing a non-inverting amplifier. This is as described by Equation (13), derived from the analysis of the circuit shown in Figure 18:

(12) $$\frac{V_{A}-V_{IN}}{R_{1}}=\frac{V_{IN}}{R_{2}}$$

(13) $$\frac{V_{A}}{V_{IN}}=(1+\frac{R_{1}}{R_{2}})$$

By setting R_2_ = R_1_ = 100 Ω [38], the voltage at node *V_A_* is demonstrated as *V_A_ = 2⋅V_IN_*. Following this stage, a high-pass filter is employed to eliminate low-frequency components from the signal. The gain value is acquired using Equation (14):

(14) $$\frac{V_{B}}{V_{A}}=\frac{{j\cdot w\cdot R}_{4}\cdot C_{1}}{{1+j\cdot w\cdot R}_{4}\cdot C_{1}}$$

We selected R_4_ = 1 kΩ and C_1_ = 100 nF. The cut-off frequency is defined by f_c,HPF_ = $\frac{1}{2\pi {\cdot R}_{4}\cdot C_{1}}$ ≃ 1.6 kHz [39].

Following the high-pass filtering, a low-pass filter is introduced. The obtained signal from the previous stage -detailed in Equation (14)—is connected to the positive differential input V_IN+_ of the MAX1448 ADC. This results in the expression for $\frac{V_{IN+}}{V_{B}},$ as shown in Equation (15):

(15) $$\frac{V_{IN+}}{V_{B}}=\frac{1}{{1+j\cdot w\cdot R}_{5}\cdot C_{2}}$$

We selected *R*_5_ = 50 Ω and *C*_2_ = 22 pF. The cut-off frequency is given by *f_c,LPF_* = $\frac{1}{2\pi {\cdot R}_{5}\cdot C_{2}}$ ≃ 145 MHz [40].

The ADC voltage level, defined as *V_IN−_ = I_COM_⋅R*_6_ = 0.25 V, is connected to the negative differential input (*V_IN−_*), where *I_COM_* = 5 mA and *R*_6_ = 50 Ω. This ensures that *V_IN+_* ≫ *V_IN−_*, where *V_IN+_* is the signal obtained after the *V_IN_* signal from the PD driver has been amplified and filtered in two stages.

To maintain synchronization between the PD driver/FPGA interface and the FPGA/LED driver interface, the same TXO crystal oscillator is connected to the MAX1448 ADC. Alternatively, the clock signal can be generated from the Xilinx Zynq 7020 FPGA [37,38,39,40,41] and connected to both the MAX5184 DAC and MAX1448 ADC by adjusting the jumper position.

The components selected for the PD driver/FPGA connection are detailed in Table 4. The datasheets of the MAX1448 ADC, the MAX4105 amplifier, and the TXO crystal oscillator can be found in [35,36,38], respectively.

#### 2.2.4. TE0720 SoC

To manage and coordinate the uplink and downlink at the Tx and Rx sides, the system requires a robust and versatile processing platform. In this context, the Trenz TE0720-03-1QFA-V1 system on a chip (SoC) module plays a pivotal role. The datasheet of the TE0720 SoC can be found in [42]. The TE0720 SoC was manufactured by Trenz and bought on https://www.trenz-electronic.de/en/ (accessed on 5 September 2024). 

The Trenz TE0720-03-1QFA-V1 system on a chip (SoC), referred to as TE0720, includes the Xilinx Zynq XA7Z020-1CLG484Q FPGA. This module features 1 GByte DDR3 SDRAM and 32 MByte QSPI flash memory. The dimensions of the TE0720 are 4 × 5 cm^2^.

**Figure 19:**
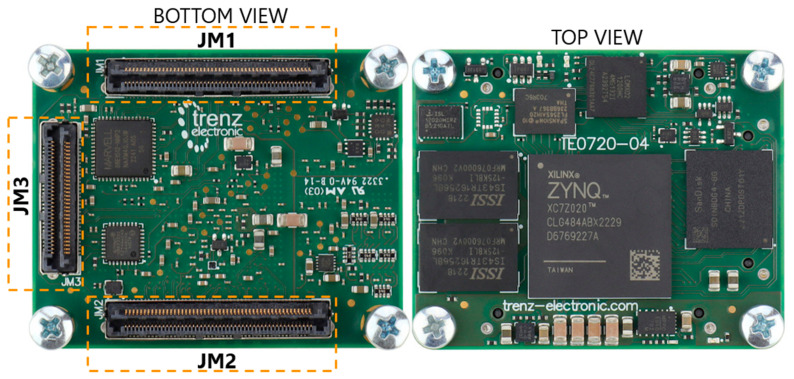
TE0720 SoC includes the FPGA Xilinx XA7z020-1CLG484Q.

The TE0720 board is equipped with three Razor Beam High-Speed Hermaphroditic Terminal/Socket Strip connectors: one 60-pin connector (labelled JM_3_) and two 100-pin connectors (labelled JM_1_ and JM_2_). These connectors are located on the underside of the printed circuit board (PCB), as depicted in Figure 19. Through these connectors, the TE0720 board interfaces with the internal interface and the external interface boards.

#### 2.2.5. Merged Internal Interfaces in a PCB

**Figure 20:**
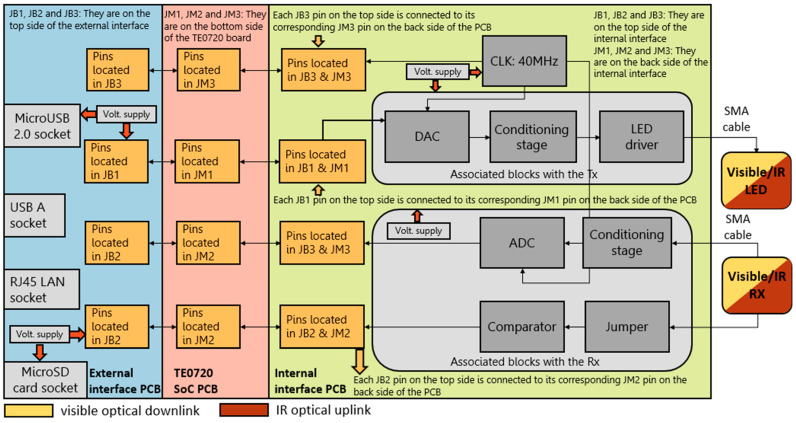
Diagram of the interaction between external interface, TE0720 SoC, and internal interface.

To minimize system noise, we decided to integrate the FPGA/LED driver interface and PD driver/FPGA interface into the internal interface. As a result, the LED driver was also included within the internal interface. This action effectively reduced the number of interacting PCBs in the system. Following this revision, the internal interface now performs three functions: (i) on the transmitter side, to ferry the output bits of the FPGA to the LED for emission, (ii) on the receiver side, to transport the analog signal received by the photodetector to the FPGA, and (iii) to link the FPGA to the external interface, which houses the input and output ports and power supply. As depicted in Figure 20, various blocks interact in the aforementioned functions, and their connectors, labelled as JB_1_, JB_2_, JB_3_, JM_1_, JM_2_, and JM_3_, are joined with their corresponding connectors on the TE0720 [42] and external interface boards. 

In reference to function (i), the interface is utilized on the LiFi router side to output the video signal on the VLC-based link and on the USB dongle to output the user requests on the IR link. For function (ii), the interface is needed on the USB dongle side to receive the video signal on the VLC-based link and on the LiFi router to receive the user requests on the IR link. With regard to function (iii), the interface is necessary to connect the FPGA to the input and output ports of the carrier board and to the appropriate power ports, which supply the required voltage and current to both the LiFi router and the USB dongle. The FPGA is embedded in the TE0720 board depicted in Figure 20.

The blocks associated with the transmitter in Figure 20 are responsible for processing the signals generated in the FPGA into an analog signal that is channeled to the LED driver. The signal introduced to the LED driver is the modulated signal emitted by the LED.

The receiver-related blocks in Figure 20 convert the analog signal from the receiver into a digital signal, which is then transferred to the FPGA for demodulation and decoding. By using a jumper, the received signal can be selected, i.e., either an OOK modulated signal or an OFDM signal [43]. This latter modulation scheme allows for the simultaneous transmission of multiple data streams at orthogonal carrier frequencies [43,44]. 

As visible in Figure 20, the LED driver module is incorporated into the internal interface within the blocks related to the Tx side.

**Figure 21:**
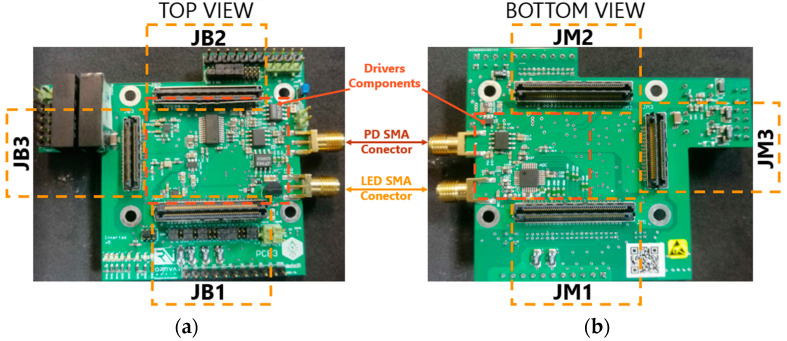
Internal interface PCB: (**a**) top view; (**b**) bottom view.

Figure 21 depicts the actual appearance of the internal interface PCB following the manufacturing and soldering process.

#### 2.2.6. External Interfaces

The aim of the external interfaces is to provide a variety of external ports such as Ethernet, USB-A, and USB-C, allowing the LiFi router and USB dongle to be connected to other communication systems accessible through Ethernet, etc. Additionally, the external interfaces provide voltage supply connectors for powering the system. 

In a nutshell, the external interfaces mainly focus on establishing a connection with external network devices and supplying power to the system. As mentioned earlier, the LiFi router external interface board operates at 12 V, whereas the USB dongle external interface board uses 5 V. Therefore, their schematic designs primarily differ in terms of the power module.

##### External Ports

With regards to the Ethernet port, the WE-MJ Femi Shielded 8P8C Ethernet connector [45] was chosen for this design. The eight signals associated with this connector are routed to the JB_1_ high-speed connector [46], enabling them to be managed by the Marvell Alaska 88E1512 Ethernet transceiver [47], which is integrated into the TE0720 board [22,42]. 

For the USB type-A port, the four signals from the universal serial bus (USB) Type-A port are routed to the pins of the high-speed connector JB_3_ [48], where they interface with the USB3320 integrated circuit (IC) [49]. This IC is housed on the TE0720 board [22,42], facilitating USB communication and functionality.

As for the JTAG, the DIGILENT 410-299 JTAG-HS3 Xilinx Tool programmer cable [50] is utilized for programming the joint test action group (JTAG) of Xilinx FPGAs. To facilitate this, a JTAG port compatible with the DIGILENT JTAG-HS3 was integrated into the external interface board, providing a connection point for the programmer cable.

For the UART, the TTL-232RG cable [51] was used to handle the universal asynchronous receiver/transmitter (UART), serving as a TTL-to-USB serial converter. To facilitate this connection, a three-pin header port [52] was integrated into the carrier board. This header offers a connection for the TTL-232RG cable, enabling communication with the FPGA through a serial port.

With regards to the microSD socket card, the implementation of a socket for reading micro secure digital (SD) cards is vital because the system’s programmable hardware, managed by the FPGA, is stored on a microSD card. Additionally, the software that runs the system application is also stored on this microSD card. If the microSD card is not inserted, the FPGA must be programmed using the JTAG programming cable. 

Concerning the high-speed connectors, the board is equipped with three Razor Beam High-Speed Hermaphroditic Terminal/Socket Strip connectors: one 60-pin connector, labelled JB_3_, and two 100-pin connectors, labelled JB_1_ and JB_2_. These connectors are located on the top side of the printed circuit board (PCB). In this manner, the signals from the Ethernet, USB Type-A, UART Tx and Rx, and JTAG ports are connected to the TE0720 board via the high-speed connectors JB_1_, JB_2_, and JB_3_.

On the other hand, the LiFi router external interface board requires a 12 V voltage supply. This voltage can be provided in two ways: (i) via an AC/DC adapter equipped with a jack power connector, or (ii) through a 12 V battery. The battery cables are connected to the external interface board using a clamp. The input voltage of the power module connects to the isolated DC/DC converter PDQE20-Q24-S12-D [53], which supports input voltages ranging from 9 V to 36 V and outputs 12 V with a current capacity of up to 1.66 A.

Similarly, the USB dongle external interface board is powered by a 5 V supply. This voltage can be provided in two ways: (i) via an AC/DC adapter with a jack power connector, or (ii) through a USB Type-C cable connected to the user’s laptop or other device.

**Figure 22:**
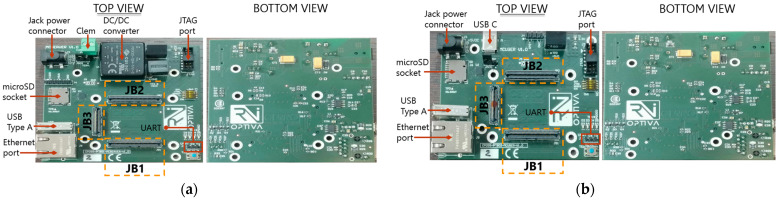
(**a**) LiFi router external interface PCB; (**b**) USB dongle external interface PCB.

Figure 22 depicts the actual physical appearance of the LiFi router external interface PCB and the USB dongle external interface PCB following the manufacturing and soldering process. The LiFi router external interface PCB measures 8.6 × 9 cm^2^, while the USB dongle external interface PCB measures 6.85 × 9 cm^2^.

### 2.3. Integration of the System’s Components

Given their symmetrical structure in the visible and infrared optical links at both ends, the LiFi router and USB dongle share the same architecture. This symmetry allows for the hardware design for both the LiFi router and the USB dongle to be identical, with the exception of the power supply voltage, which is 12 V for the LiFi router and 5 V for the USB dongle. This power supply difference influences all the system power modules, LED drivers, and interfaces. 

**Figure 23:**
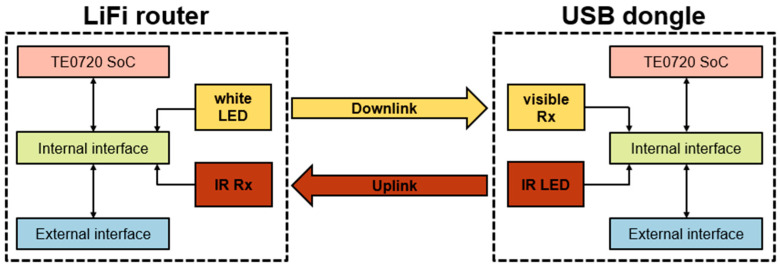
Block diagram of all PCBs that are part of the system.

Figure 23 below presents a block diagram of the PCBs interacting within the system.

**Figure 24:**
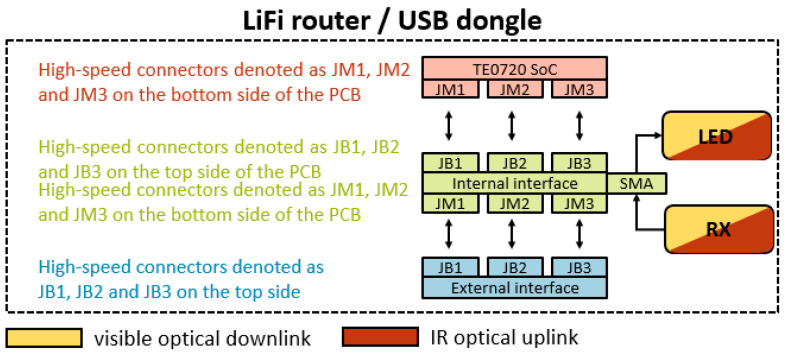
Schematic connection of the components of the system for LiFi router and USB dongle.

Figure 24 displays the connections among the PCBs that constitute the LiFi router and/or the USB dongle. The TE0720 SoC, internal interface, and external interface boards are stacked and connected via high-speed connectors [46,48]. The TE0720 SoC board has high-speed connectors on its back side, while the external interface board features them on its top side. Conversely, the internal interface PCB incorporates high-speed connectors on both its top and bottom sides.

The high-speed connectors on the top side of the PCBs are labelled as JB_1_, JB_2_, and JB_3_, whereas those on the back side are labelled as JM_1_, JM_2_, and JM_3_, as shown in Figure 24.

The JB_1_, JB_2_, JM_1_, and JM_2_ connectors each have 100 pins [46], whereas the JB_3_ and JM_3_ connectors have 60 pins each [48]. The previously mentioned high-speed connectors are the Razor Beam High-Speed Hermaphroditic Terminal/Socket Strip connectors.

### 2.4. LiFi Router PCB Connections

**Figure 25:**
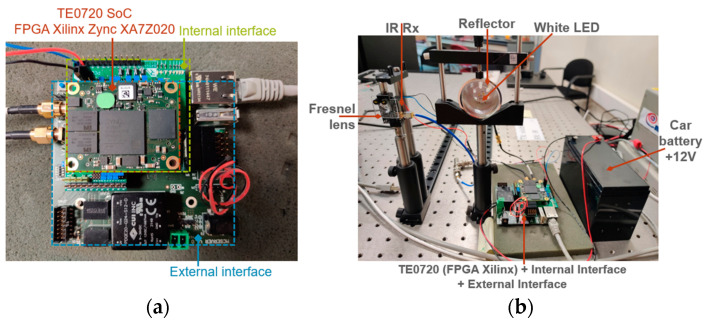
(**a**) PCBs stack of TE0720 SoC, internal and external interfaces; (**b**) LiFi router.

The LiFi router is composed of five distinct PCBs. The visible LED board, which is equipped with two white LEDs, links to the system via an SMA cable connected to the internal interface board. In a similar manner, the IR receiver board links to the system using another SMA cable connected to the internal interface board. The board stack includes the TE0720 SoC module, the internal interface board, and the external interface board, as depicted in Figure 25a. Additionally, the LiFi router, which is powered by a 12V battery, is shown in Figure 25b. 

### 2.5. USB Dongle PCB Connections

**Figure 26:**
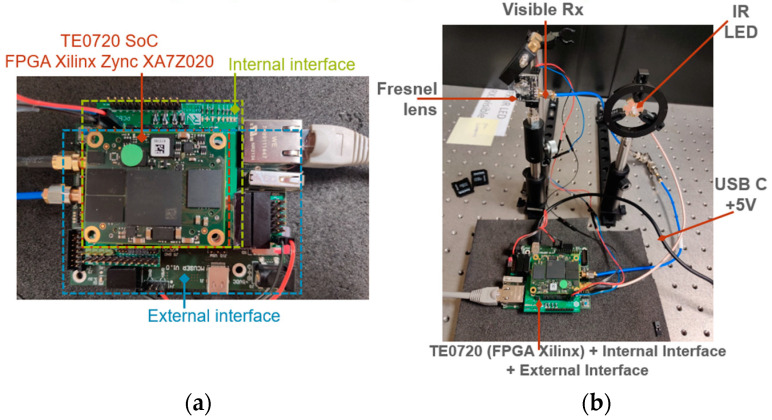
(**a**) PCBs stack of TE0720 SoC, internal and external interfaces; (**b**) USB dongle.

The USB dongle is made up of five distinct PCBs. The IR LED board, which carries the IR LED, is connected to the system via an SMA cable to the internal interface board. Similarly, the visible receiver board is connected to the system through another SMA cable to the internal interface board. The board stack includes the TE0720 SoC module, the internal interface board, and the external interface, as depicted in Figure 26a. It is important to note that the user device is powered by 5 V via the USB-C cable, which connects to a laptop, as illustrated in Figure 26b. 

The signal received in the USB dongle can be connected to various devices for simultaneous UHD video playback on different devices such as a laptop, a 4K TV, and an XBOX 360, as shown in https://youtu.be/wNDX3lWwILc (accesed on 5 September 2024). This video can be downloaded in the Supplementary Materials section. As such, this system functions as a single-input multiple-output (SIMO) set-up.

### 2.6. Experimental Set-Up and Testing Procedures

**Figure 27:**
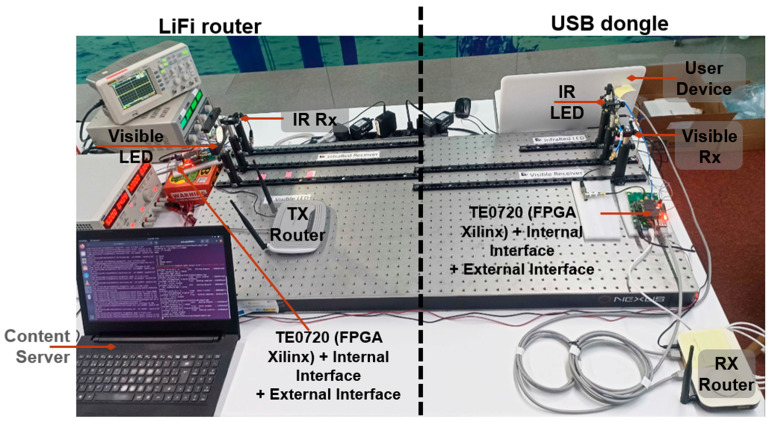
Set-up carried out to test every module of the system.

The subsequent sections detail the tests conducted to independently validate the Tx block, the Rx block, and the combined Tx-Rx within the same PCB operating in a closed loop. To carry out these tests was implement the set-up shown in Figure 27.

#### 2.6.1. Testing Procedure for the Transmitter Block

**Figure 28:**
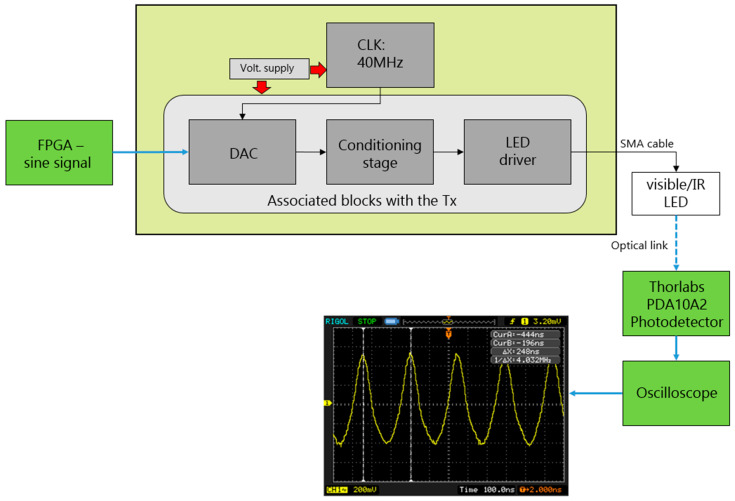
Scheme of the testing procedure for the transmitter block.

In this section, we detail the procedure used to verify the proper functioning of the transmitter block in the system, as illustrated in Figure 28. This subsystem is situated in the internal interface PCB.

**Figure 29:**
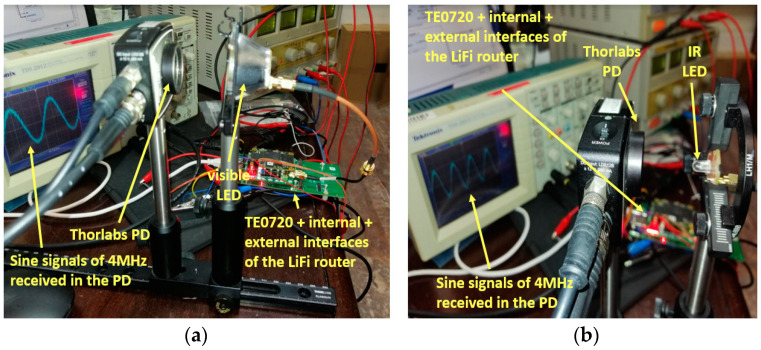
Tests carried out in the transmitter block utilizing a (**a**) visible LED and (**b**) IR LED.

The testing process involved generating a 4 MHz sine signal using the FPGA, as shown in Figure 29. This signal was transmitted via an LED to the Thorlabs PDA10A2 PD [28], which spans a spectral range from 200 nm to 1100 nm and possesses a bandwidth of 150 MHz. The PD was connected to a Tektronix 2012 oscilloscope to observe the received signal, as depicted in Figure 28.

These tests were conducted using both the visible LED and the IR LED, as the system’s uplinks and downlinks employ visible and infrared channels.

Figure 29a illustrates the set-up for testing the transmitter block, using the LUW CN7N-KYLX-EMKM OSRAM LED, which was bought on https://eu.mouser.com/ (accessed on 5 September 2024). The distance between the visible LED and the Thorlabs PDA10A2 PD was 7 cm. The Tektronix 2012 oscilloscope screen in Figure 29a shows the signal received by the PD, which measured 4 MHz—the same frequency as the sine signal transmitted through the transmitter block.

Likewise, Figure 29b depicts the test set-up of the transmitter block using the HSDL-4250 Lite-On IR LED. The distance between the infrared LED and the Thorlabs PDA10A2 PD was 6 cm. The oscilloscope screen shown in Figure 29b indicates that the PD-received signal frequency was 4 MHz, identical to the frequency of the sinusoidal signal transmitted through the transmitter block.

#### 2.6.2. Testing Procedure for the Receiver Block

**Figure 30:**
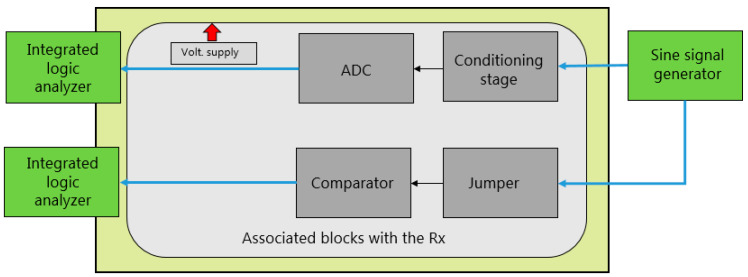
Implemented test to validate the receiver block.

To validate these subsystems, tests were conducted by connecting the RIGOL DG5102 arbitrary function generator to the input of the signal-conditioning module and monitoring the 10 output bits of the ADC through the Integrated Logic Analyzer (ILA) programmed in the FPGA. Additionally, the comparator block was tested by activating it via the jumper on the board and observing the output bit through the ILA, as illustrated in Figure 30.

For the tests associated with the conditioning stage and the ADC, signals of various frequencies were sent through the signal generator, and the ADC’s output was monitored via the ILA programmed in the FPGA. Similarly, for the comparator block tests, signals of different frequencies were sent through the signal generator, and the comparator output was observed using both the ILA and the Tektronix 2012 oscilloscope.

**Figure 31:**
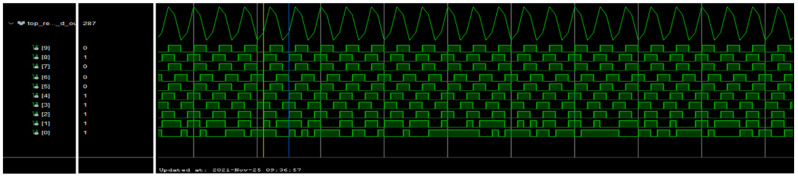
ILA captured at the ADC’s outputs.

For instance, Figure 31 presents a capture from the ILA, showcasing a 10 MHz signal input delivered from the signal generator.

#### 2.6.3. Testing Procedure for the Transmitter and Receiver Block Located in the Same PCB

**Figure 32:**
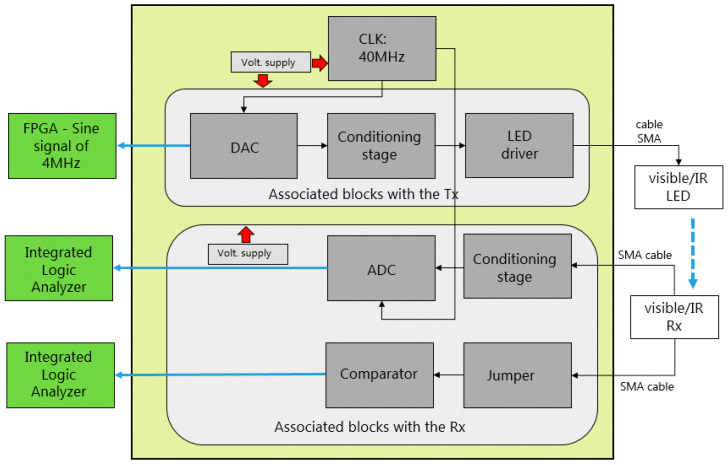
Test carried out to validate the Tx block and Rx block working in closed loop.

The validation of the transmitter and receiver blocks on the same board involved the transmission of a 4 MHz sine signal, generated by the FPGA. Subsequently, the outputs from the ADC and the comparator were observed using the ILA, as shown in Figure 32.

### 2.7. Deployment of the System in a Van

The overarching aim of this study is to implement the developed VLC system for use inside a van, specifically a Transit Kombi van in this case.

**Figure 33:**
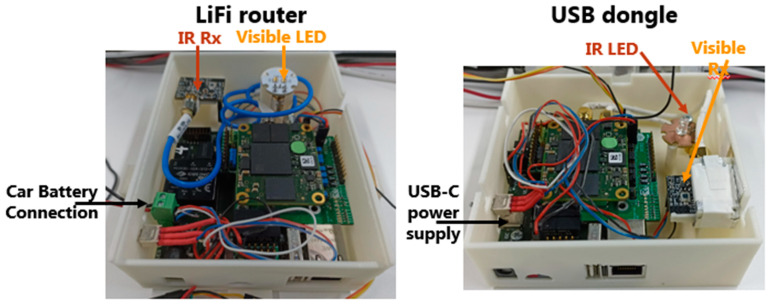
LiFi router and USB dongle packed in boxes.

To fulfill the objective outlined in this work, each component of the LiFi router and the USB dongle was housed in dedicated plastic boxes, specifically designed for deployment within the van, as illustrated in Figure 33. Additionally, the VLC system was tested at a distance of 72.5 cm, representing the distance between the LiFi router and the USB dongle within the van.

**Figure 34:**
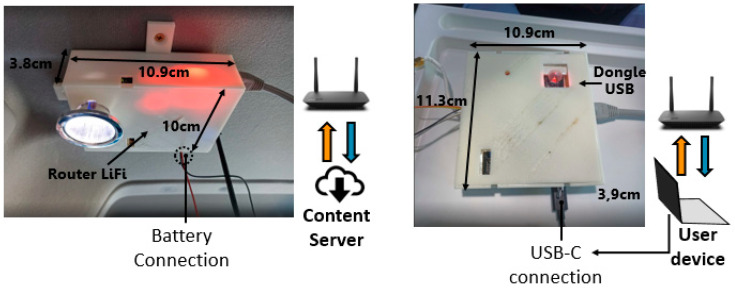
The VLC system developed was deployed using a Ford Transit model van.

The LiFi router was installed on the interior roof of the van, as portrayed in Figure 34. Simultaneously, the USB dongle was situated on a portable table adjacent to the user’s laptop for UHD video viewing, as depicted in Figure 34. The LiFi router is powered by a 12 V battery, while the USB dongle is powered supplied by a USB-C cable connected to the laptop. 

**Figure 35:**
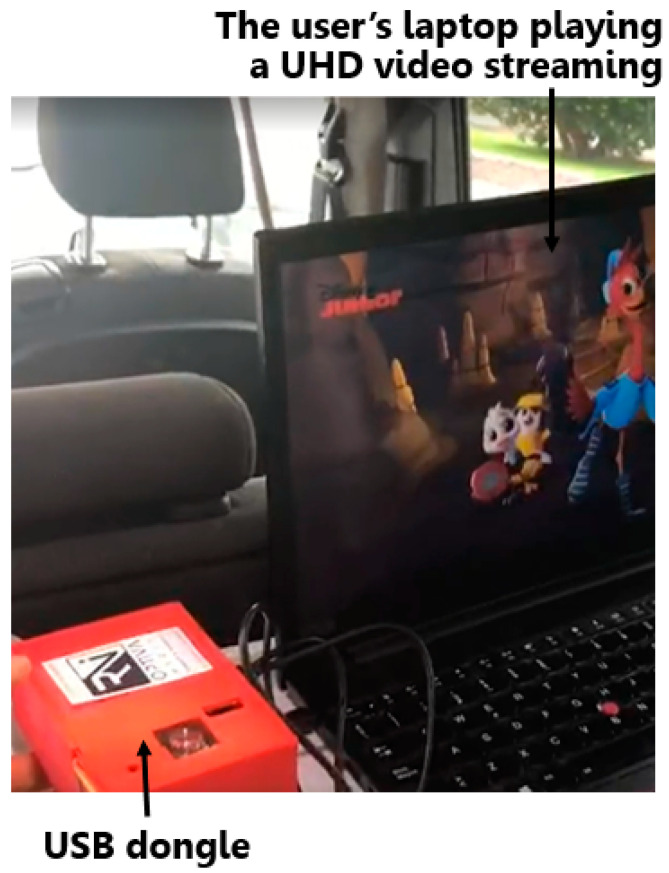
The user’s laptop playing a UHD video streaming due to the implemented USB dongle.

Figure 35 shows a potential use case scenario for the optical transceivers and optoelectronics interfaces developed in this work, where UHD video is streamed through the reading lamps of a passenger van.

## 3. Results

### 3.1. Supply Voltages and Current Consumption

To guarantee the optimal functioning of the bidirectional system with visible and IR capabilities, the LiFi router is powered with a 12 V supply, consuming 920 mA of current. The supply voltage of the USB dongle is 5 V, and the current consumption is 1.12 A.

### 3.2. Energy Harvesting Performance

An ALLPOWER 200W solar panel was located on the roof of the passenger van to supply power to the USB dongle and the user’s laptop. This solar panel is capable of delivering up to 2 A and 5 V under a luminous flux of 80,000 lx of sunlight. As the USB dongle’s current consumption is 1.12 A, the USB dongle was completely self-powered in the previous conditions of luminosity. Moreover, a 26,800 mAh power bank battery was connected to the solar panel to store the generated power. In this set-up, the outputs of the power bank battery were connected to the laptop and the USB dongle. It took 13.4 h to charge the battery with the power generated by the solar panel, and the battery life was 22.3 h. These values allowed us to verify that the USB dongle and the laptop were self-powered by the solar panel. Therefore, the user could watch UHD videos without worrying about the power supply of the laptop or the USB dongle. It is important to clarify that without the energy harvesting capabilities, the USB dongle and the user’s laptop had an autonomy of 22.3 h. 

### 3.3. Electro-Optical Measurements of the Optical Links

The electro-optical measurements for the visible and IR links included the bandwidth, illumination level for the visible link LED, signal-to-noise ratio (SNR), bit rate, bit error rate (BER), packet error rate (PER), and the reach of the optical links. These measurements were conducted in the system shown in Figure 34, where the system was deployed within the passenger van. The maximum optical link length achieved in the passenger van was 72.5 cm, while the maximum optical link length in the set-up shown in Figure 27 was 102.5 cm. The USB dongle within the van was positioned in the passenger seat, where sunlight exposure did not have significant effects on the PD. 

#### 3.3.1. Electro-Optical Measurements of the Visible Link

This section presents the electro-optical characterization of the system’s visible link. The measured parameters encompassed the bandwidth, illumination level of the ultra-white LED, minimum signal-to-noise ratio (SNR_min_) to ensure UHD video transmission, bit rate, bit error rate (BER), packet error rate (PER), and length of the visible optical link. 

##### Bandwidth (BW) of the Visible Link

The system’s bandwidth is constrained by the white LED used as the video transmitter. This LED has been electrically and optically characterized. The optical measurements conducted with the Thorlabs PDA10A2 photodetector, which has a bandwidth of 150 MHz, revealed that the LED’s bandwidth was 1.8 MHz. As this bandwidth was deemed insufficient for UHD video transmission, a pre-equalizer stage was engineered into the LED driver to increase it to 14.45 MHz. The frequency behavior of the signal emitted by the LED was assessed using a RIGOL DSA 815 spectrum analyzer, bearing a bandwidth of 1.5 GHz. Although the equalized visible LED bandwidth reached 14.45 MHz, the visible PD driver achieved 11.66 MHz of bandwidth, which set a limit on the downlink bandwidth. In essence, the bandwidth of the downlink was 11.66 MHz. This value surpasses the requirement of 10 MHz, as the system transmits information at 10 MHz. This frequency ensures an exact number of samples per symbol, as it is a submultiple of the 40 MHz sampling frequency of the A/D and D/A converters.

##### Illumination Level

The illumination level of the LUW-CN7N-KYLX-EMKM OSRAM LED, operating at a current of 170mA in the set-up shown in Figure 34, was measured using the MAVOLUX 5032C USB luxmeter, obtaining a value of 75,000 lx. 

##### Signal-to-Noise Ratio (SNR) of the Visible Link

A minimum signal amplitude of 200 mV_pp_ at the receiver’s output is necessary to ensure the correct performance of the visible downlink. This value was experimentally determined in the set-up portrayed in Figure 34.

The noise amplitude measured at the receiver’s output was 45 mV_pp_, and this measurement was carried out without transmitting any data from the LiFi router. 

The SNR_min_ was calculated using the aforementioned values, yielding a result of 10.74 dB. This SNR_min_ was achieved at a 72.5 cm distance between the visible LED and the visible PD within the passenger van. Meanwhile, a similar SNR value was observed in the set-up depicted in Figure 27 at a 110 cm distance between the visible LED and the visible PD, where both were positioned in the indoor set-up. 

#### 3.3.2. Data Transmission Parameters of the Visible Link

This section examines the data transmission parameters achieved by an implementation of a UHD video transmission using the electro-optical interface proposed in this work to send the data through visible light. This implementation is only one of many applications where the interface can be used, and the parameters depend not only on the optoelectronics interfaces but also on the modulation and coding formats, which depend on the VHDL development. 

##### Bit Rate of the Visible Link

To transmit UHD-quality video, a minimum transmission speed of 5 Mbps is required, depending on the video encoding. Throughout the development of this system, various modulations and encodings were tested. To measure the transmission speed, the VLC link was configured to operate at its maximum speed, and the time taken by the VLC system to send 1 MB was recorded. The results are presented in Table 5. These results demonstrate that UHD-quality video can be transmitted at the minimum required speed, provided the video is correctly encoded. The correction factor R = 1 indicates that all transmitted bits are information bits. In contrast, $R=\frac{p}{q}$ indicates that for every q bit sent, p bits are information and (p−q) bits are redundancy bits used for error correction. 

##### Bit Error Rate (BER) and Packet Error Rate (PER) of the Visible Link

To ensure perfect fluidity of multimedia content, it is essential to achieve zero bit errors and zero packet errors, meaning no packet losses. To evaluate the system’s performance regarding these criteria, a test was conducted involving the consecutive transmission of 256 random packets, with each packet containing 4096 bytes. In total, 8,388,608 random bits were transmitted during this test. These tests were performed at various distances ranging from 62.5 cm to 102.5 cm, as detailed in Table 6.

At a distance of 102.5 cm, the system was capable of receiving almost all packets, achieving a very satisfactory packet error rate (PER), although it was not possible to evaluate this numerically. As expected, the bit error rate (BER) increased with the distance. Despite this, all the BER values obtained were less than $3.8\times 10^{-3}$ [54,55], which is known as ‘error free’ in optical transport networks, allowing for the application of Forward Error Correction (FEC) techniques at all distances. When the BER is below this threshold, FEC techniques can be employed to correct errors, preventing any data loss. Conversely, if the BER surpasses this value, FEC techniques are unable to correct the errors, leading to data loss. The effect of this on transmission depends on the type of data being transmitted.

By implementing an error correction system, the bit error rate was minimized, ensuring the optimal performance of the VLC system for video transmission with the necessary quality.

##### Optical Link Length of the Visible Link

The maximum distance at which that the system could maintain UHD video without losing was measured using an optical table and rails, which allowed for increasing the distance between the receiver and transmitter without altering other factors such as the direction of the light beam or the distance between the receiver and the lens, as shown in Figure 34.

Measurements were taken of the number of errors per packet at various distances. As the receiver moved farther from the transmitter, more errors appeared in the packets. This result was expected because the noise level at the receiver remained nearly constant while the signal intensity decreased with increasing distance.

To achieve a distance of 110 cm in the set-up depicted in Figure 27, the maximum without video errors, an error correction factor of $R=\frac{1}{2}$ was required, as the number of errors per packet exceeded 30, which could not be corrected using other encoding methods. For distances less than 92.5 cm, an error correction factor of $R=\frac{2}{3}$ was used, addressing the errors per packet exceeding 15. For distances less than 82.5 cm, an error correction factor of $R=\frac{3}{4}$ was sufficient, where the errors per packet were fewer than 5.

All these measurements were conducted at the center of the beam to obtain the characteristics of the system in optimal conditions. It is clear then that when the detector moves from the center of the beam, the performance in terms of data transmission is degraded, as demonstrated in [56].

#### 3.3.3. Electro-Optical Measurements of the IR Link

This section presents the electro-optical characterization of the system’s IR link. The measurements included the bandwidth, signal-to-noise ratio (SNR), bit rate, bit error rate (BER), and length of the IR optical link.

##### Bandwidth (BW) of the IR Link

Despite the equalized IR LED bandwidth being more than 20 MHz, the IR PD driver managed to achieve a bandwidth of 12.27 MHz, which constrains the bandwidth of the uplink. In summary, the bandwidth of the uplink is 12.27 MHz. This value exceeds the requirement of 10 MHz, as the system transmits information at 10 MHz. This frequency ensures an exact number of samples per symbol, as it is a submultiple of the 40 MHz sampling frequency of the A/D and D/A converters.

##### Signal-to-Noise Ratio (SNR) of the IR Link

Similar to the visible link, the received signal level in the infrared link must be at least 200 mV_pp_, with a noise level of 45 mV_pp_. These signal and noise levels yield a minimum signal-to-noise ratio (SNR_min_) of 10.74 dB for the infrared link.

#### 3.3.4. Data Transmission Parameters of the IR Link

This section examines the data transmission parameters achieved in the IR link (uplink).

##### Bit Rate of the IR Link

In order to measure the transmission speed, the infrared link was configured to operate at its highest possible speed, and the time taken by the IR system to send 1 MB was recorded. Using OOK 8b10b modulation and coding, the system achieved a maximum rate of 1.91 MB/s, which is equivalent to 15.25 Mbps.

##### Bit Error Rate (BER) of the IR Link

Employing OOK modulation for the IR link resulted in no detectable errors within the packets. This is because the average power level was maintained constant, meaning if the power level of a symbol ‘1’ decreased, the level of all subsequent symbols also decreased, resulting in packet errors. To mitigate this, the packet size was set to 1 byte for command transmission in the IR channel, reducing the overall probability of errors as long as the amplitude level of the symbols remained distinguishable.

##### Optical Link Length of the IR Link

The maximum distance for maintaining command reception was measured using an optical table and rails, allowing for the distance between the receiver and transmitter to be varied without altering other factors such as the direction of the IR light beam or the distance between the receiver and the lens, as shown in Figure 34.

Commands were successfully received without errors at distances ranging from 60 cm to 110 cm. For distances from 72.5 cm up to 110 cm, the set-up depicted in Figure 27 was employed. Distances beyond 110 cm were not measured, as the video transmission link’s limit is 110 cm, and both the visible and infrared links are part of an integrated system that must operate at the same distances.

## 4. Discussion and Conclusions

This work thus illustrates that utilizing a VLC system to facilitate the transmission of UHD content in commercial vehicles is feasible and scalable for mass transit applications, as demonstrated in the following link: https://www.youtube.com/watch?v=GH5htUZf-Uw (accessed on 5 September 2024). This video can be downloaded in the Supplementary Materials section.

The implemented system comprises two parts. The first part is the ‘LiFi router’, which is made up of a stack of three boards, a visible LED, and an infrared receiver. It operates on a supply voltage of 12 V with a current draw of 920 mA. There are two power sources it can utilize: (i) a 12 V AC/DC adapter with a jack power connector or (ii) a 12 V battery. The ultra-white LED, driven by the implemented driver on one of the boards, achieves a bandwidth of 14.45 MHz, while the IR PD driver attains a bandwidth exceeding 12.27 MHz.

The second part is the USB dongle, which also consists of a stack of three boards, an IR LED, and a visible-light receiver. Operating with a 5 V supply voltage and drawing 1.12 A, this part can be powered in two ways: (i) via a 5 V AC/DC adapter with a jack power connector or (ii) via a USB Type-C connector. The infrared LED, accompanied by the implemented driver, achieves a bandwidth exceeding 20 MHz, whereas the visible photodiode (PD) driver, powered by the interface board at +5 V and −5 V, attains a bandwidth surpassing 11.66 MHz.

Additionally, the LED driver is designed for high-power white LEDs with a power consumption of approximately 1.5 W, replicating the reading lamps commonly found in mass transit. The first two stages of the driver consist of equalization amplification circuits designed to enhance the bandwidth of the LED. Each of these stages incorporates phase-advanced equalizers, resulting in a bandwidth of 14.45 MHz, marking a significant improvement (almost a decade) from the initial bandwidth of 1.8 MHz. The final stage encompasses a current source, comprising a voltage-biased N-MOSFET transistor that superimposes the modulated signal on the DC level necessary for the LED to achieve a luminous intensity of 75,000 lx.

Conversely, the developed PD driver, comprising three gain stages, attains a bandwidth of 11.66 MHz for the visible PD driver and 12.27 MHz for the IR PD driver. While these are modest values, they are sufficient to transmit UHD content as required.

In summary, the bandwidth of the visible downlink is 11.66 MHz, while the IR uplink achieves a bandwidth of 12.27 MHz. In [10], a bandwidth of 8 MHz and a link distance of 8 m were reported. However, that system employed an APD as the photodetector. Considering that APDs require high bias voltages such as 65 V, and given that the receiver needs to be powered by a 5 V supply, using an APD is not viable.

To avoid potential spurious data, the visible optical link was configured to operate at a speed of 10 MHz, a submultiple of the 40 MHz sampling frequency determined by the ADC and DAC. Moreover, the maximum optical length achieved is 110 cm, accomplished by using a Fresnel lens at the receiver side with a focal length of 10 mm and a diameter of 13 mm. The system’s noise level amplitude is approximately 45 mV_pp_.

Likewise, the infrared optical link operates at 10 MHz, in harmony with the 40 MHz sampling frequency. It also achieves an operating distance of 110 cm, utilizing the same Fresnel lens. The system’s noise level amplitude is approximately 45 mV_pp_.

To ensure the system’s correct performance, the minimum signal-to-noise ratio (SNR) for both optical links is set at 10.74 dB.

The visible optical channel was modulated using a multicarrier modulation scheme, with each carrier modulated using Quadrature Phase Shift Keying (QPSK). This set-up achieved a BER of 3.3259×10^−5^ at a distance of 72.5 cm between the LED and the PD, both situated in the passenger van, reaching a bit rate of 11.25 Mbps.

The infrared link employs OOK with 8b10b encoding. As the data integrity requirements are less stringent than in the downlink, any packet lost due to interference is simply retransmitted, making this a suitable option. This uplink set-up reached a maximum data rate of 15.25 Mbps.

In conclusion, a system capable of streaming UHD video was successfully implemented using the hardware design of the optical transceivers and optoelectronic interfaces executed in this work. Specifically, this system achieved a bit rate of 15.25 Mbps for OOK and 11.25 Mbps for QPSK within the OFDM scheme. For context, the system reported in [7] achieved up to 2 Mbps for video transmission using a red LED and reached an optical link of 50 cm, which is insufficient for UHD video transmission. In the work documented in [8], a data transmission speed of 0.986 Mbps was achieved over a distance of 5 m. However, a VLC system with this bit rate cannot support the UHD transmission rate targeted in this study.

In [11], a maximum bit rate of 0.415 Mbps was reported, with video qualities ranging from 480p to 720p -far from the UHD focus of this work -. In [12], a system was developed with a bit rate of 7.14 Mbps over an optical link of 6.7 cm, while the optical link achieved in this work is 110 cm. Although [13] displayed an optical link of 100 cm, the bit rate reached was only 0.115 Mbps, inadequate for UHD video transmission. An optical link of 5 m with a bit rate of 0.115 Mbps was exhibited in [14], and [9] presented an optical link distance of 6 m with a bit rate of 0.986 Mbps. Both of these instances fall short of the requirements for UHD video transmission.

Although [15] reported a UHD video transmission with a bit rate of 100 Gbps, the system utilized high-power lasers for Free Space Optical (FSO) Communication links. It is well known that high-power lasers are prohibited in environments where people are present due to the risk of inducing cornea injuries [5] and skin burns [6]. Therefore, the system outlined in [15] is unsuitable for user applications. Consequently, our proposed VLC system, capable of transmitting UHD video, remains the foremost option in the current state of the art for user applications. It is also compliant with the pre-existing lighting infrastructure in public transportation settings. A proof of concept was carried out by deploying the system in a passenger van, where it was successfully tested. Finally, a solar panel situated on the van’s roof was employed to power the user’s side, namely, the USB dongle and the user’s laptop, through a power bank battery. With this set-up, the user’s side was completely self-powered, charging the battery in 13.4 h under a luminous flux of 80,000 lx, while the battery life lasted 22.3 h.

The terms abbreviation list can be found in the following Table 7.

## Figures and Tables

**Table 1:** Components of the LED driver.

Input		Equalizer 1		Amplification Stage 1		Equalizer 2		Amplification Stage 2		Current Source	
R_5_	200 Ω	R_1_	3.9 kΩ	R_6_	20 kΩ	R_3_	1 kΩ	R_10_	1 kΩ	R_13_	10.7 kΩ
C_3_	1 pF	C_1_	22 pF	R_7_	1 kΩ	C_2_	25 pF	R_11_	12 kΩ	R_14_	5 kΩ
V_in_(AC)	0.8V_pp_	R_2_	1 kΩ	R_8_	500 Ω	R_4_	50 Ω	R_12_	1 kΩ	R_15_	5.5 Ω
V_in_ (DC)	0 V			C_4_	56 nF	C_6_	56 nF	C_6_	56 nF	TRT	TN0110N3
				Ampl	LTC6268-10			Ampl	LTC6268-10	V_dd_	12 V
				R_9_	200 Ω						
				C_5_	not mounted						

**Table 2:** Components of the PD driver.

Amplification Stage 1		Amplification Stage 2		Amplification Stage 3	
R_1_	5 kΩ	R_4_	200 Ω	R_8_	900 Ω
R_2_	5 kΩ	C_5_	100 nF	R_9_	100 Ω
C_4_	56 nF	R_7_	1 kΩ	Amplifier	LTC6253
R_7_	15 kΩ	R_5_	16 kΩ		
C_3_	0.25 pF	R_6_	1 kΩ		
Amplifier	LTC6253	C_6_	100 nF		
		Amplifier	LTC6253		

**Table 3:** Components of the FPGA/LED driver connection.

R_1_	402 Ω	R_2_	1 kΩ	DAC	MAX5184
R_3_	402 Ω	R_4_	1 kΩ	Amplifier	MAX4105
				Oscillator	TXO crystal 40 MHz

**Table 4:** Components of the PD driver/FPGA connection.

R_1_	100 Ω	R_3_	1 kΩ	C_2_	22 pF
R_2_	100 Ω	R_4_	1 kΩ	R_6_	50 Ω
C_1_	100 nF	R_5_	50 Ω	C_3_	22 pF
ADC	MAX1448	Amplifier	MAX4105	C_4_	100 nF
Oscillator	TXO crystal 40 MHz				

**Table 5:** Bits rates of the VLC link.

Modulation and Codification	MB/s	Mbps
OOK (8b10b)	1.91	15.25
OFDM (BPSK, R = 1)	0.71	5.69
OFDM (QPSK, R = 1)	1.41	11.25
$\mathrm{OFDM}\;(\mathrm{QPSK},\;R=\frac{5}{6}$)	1.23	9.83
$\mathrm{OFDM}\;(\mathrm{QPSK},\;R=\frac{3}{4}$)	1.05	8.44
$\mathrm{OFDM}\;(\mathrm{QPSK},\;R=\frac{1}{2}$)	0.71	5.69

**Table 6:** BER and PER measured for the VLC link.

Distance (cm)	BER	Errors per Packet	Number of Packets with Errors	PER
62.5 ^(1)^	$1.7166\times 10^{-5}$	3	48	0.1875
72.5 ^(1)^	$3.3259\times 10^{-5}$	3	93	0.3632
82.5 ^(2)^	$7.3909\times 10^{-5}$	5	124	0.4843
92.5 ^(2)^	$1.5258\times 10^{-4}$	8	160	0.625
102.5 ^(2)^	$7.3432\times 10^{-4}$	28	220	0.8593

(1) Measurements carried out in the set-up in the passenger van depicted in Figure 34. (2) Measurements carried out in the set-up depicted in Figure 27.

**Table 7:** Terms abbreviation list.

AC	Alternating Current	PCB	Printed Circuit Board
ADC	Analog-to-Digital Converter	PD	Photodiode
AlGaAs	Aluminum Gallium Arsenide	PER	Packet Error Rate
APD	Avalanche Photodiode	PHY	Physical
ARM	Advanced RISC Machine	PIN	Positive–Intrinsic–Negative
BW	Bandwidth	QSPI	Quad Serial Peripheral Interface
DAC	Digital-to-Analog Converter	QPSK	Quadrature Phase Shift Keying
DC	Direct Current	RF	Radio-Frequency
DDR3	Double Data Rate version 3	Rx	Receiver
FPGA	Field-Programmable Gate Array	RISC	Reduced Instruction Set Computer
FSO	Free Space Optical	SD	Secure Digital
GBW	Gain-Bandwidth	SDRAM	Synchronous Dynamic Random-Access Memory
IC	Integrated Circuit	SIMO	Single-Input Multiple-output
IFFT	Inverse Fast Fourier Transform	SMA	Subminiature version A
ILA	Integrated Logic Analyzer	SNR	Signal-to-Noise Ratio
IoT	Internet-of-Things	SoC	System On a Chip
IR	Infrared	TTL	Transistor–Transistor Logic
JTAG	Joint Test Action Group	TV	Television
LAN	Local Area Network	Tx	Transmitter
LED	Light-Emitting Diode	UART	Universal Asynchronous Receiver–Transmitter
LiFi	Light Fidelity	UHD	Ultra-High Definition
LoRa	Long-Range	USB	Universal Serial Bus
MAC	Medium Access Control	VLC	Visible-Light Communication
OFDM	Orthogonal Frequency-Division Multiplexing	WAN	Wide Area Network
OOK	On–Off Keying	WiFi	Wireless Fidelity
OWC	Optical Wireless Communication		

## Data Availability

For further data information, please contact to correspondence author.

## Supplementary Materials

The following supporting information can be downloaded at: https://www.mdpi.com/article/10.3390/s24175829/s1, Videos S1: Developed system in the lab, and deployed in a passenger van.
